# Supplementary material for: Energy-selective confinement of fusion-born alpha particles during internal relaxations in a tokamak plasma
Source: Nat Commun. 2022 Jul 8;13:3941. doi: 10.1038/s41467-022-31589-6 (PMC9270425; doi:10.1038/s41467-022-31589-6)
Supplement: Supplementary file 1 — Supplementary Information [file 41467_2022_31589_MOESM1_ESM.pdf]

Supplementary Information for "Energy-selective confinement of fusion-born alpha particles during internal relaxations in a tokamak plasma"

by A. Bierwage, K. Shinohara, Ye.O. Kazakov, V.G. Kiptily, Ph. Lauber, M. Nocente, Ž. Štancar, S. Sumida, M. Yagi, J. Garcia, S. Ide and JET Contributors

This document contains detailed information about the simulation setup, more complete views of the results shown in the main paper, and additional results of parameter scans and sensitivity tests.

List of Supplementary Notes

1. Simulation scenario design 1
2. Fourier harmonics of the internal kink mode 2
3. Comparison with full-domain simulation: Mode evolution, fast alphas & peripheral islands 3
4. Guiding center orbit topology 3
5. Particle redistribution in velocity space 5
6. Gyroaveraging 6
7. Parallel electric field 7
8. Safety factor profile scan 7

Supplementary References 10

JET Contributors 12

Supplementary Note 1. Simulation scenario design

The simulation setup is described in some detail in [1], where we focused on the modeling of an alpha particle distribution. Here we describe in more detail the model for the bulk plasma in our sawtooth crash simulations.

The profiles of both the fast ion pressure and the MHD pressure are based on a reconstruction of a JET plasma using the code TRANSP-EFTP as reported in [2]. The MHD pressure in these TRANSP-EFTP simulations included the fast ion component. We have subtracted that part as shown in Supplementary Fig. 1 and recomputed the equilibrium using only the estimated pressure of the bulk plasma.

By reducing the pressure, we were able to suppress resistive interchange modes that would otherwise overwhelm the internal kink mode in our visco-resitive MHD simulations with Lundquist and Reynolds numbers $S = \mathrm{Re} = 10^6$. Here, $\mathrm{Re} = v_{\mathrm{A0}} R_0/\nu$ with viscosity ν, major radius R_0 and on-axis Alfvén speed v_{A0}. $S = \tau_\eta/\tau_{\mathrm{A0}}$ is the ratio of the resistive and toroidal Alfvénic time scales, $\tau_\eta = \mu_0 R_0^2/\eta$ and $\tau_{\mathrm{A0}} = R_0/v_{\mathrm{A0}} \approx 0.4\,\mu\mathrm{s}$, where η is the electrical resistivity and $\mu_0 = 4\pi \times 10^{-7}\,\mathrm{H\,m^{-1}}$ the vacuum permeability. A certain amount of MHD pressure can, however, be beneficial as it seems to have a numerically stabilizing effect: test runs initialized with a zero-pressure MHD equilibrium terminated abnormally during the nonlinear saturation stage. We suspect that compressible MHD effects help to regularize the dynamics, for instance by reducing fine structures in the reconnection layer that may otherwise cause numerical problems. Possible mechanisms realizing this include secondary interchange modes and magnetosonic wave propagation. The value of the specific heat ratio that controls compressibility in our MHD simulations was fixed at $\Gamma = 5/3$.

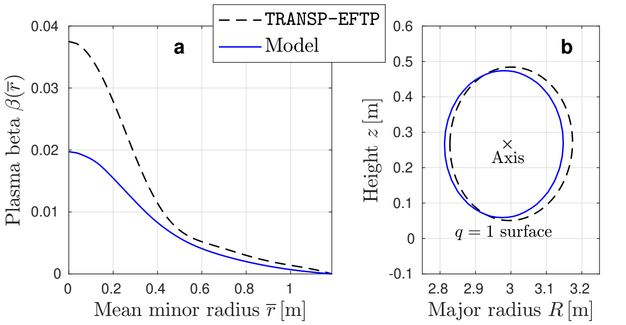

Supplementary Fig. 1. (**a**) Pressure profile used for MHD force balance calculations. The dashed curve is the pressure profile predicted by an integrated TRANSP-EFTP simulation for JET pulse 95679 at 50.22554 seconds, which includes the contribution of fast ions from beams and RF heating, but not the effect of sawteeth. The solid curve is our model, which includes only the estimated bulk plasma component. (**b**) Excluding the fast ion component from the MHD pressure reduces the Shafranov shift, so that the $q = 1$ surface shifts inward in R as shown here for the case with $q_0 = 0.98$.

As stated in the text of the main article, we treat the safety factor profile $q(\psi_{\mathrm{P}})$ as a free parameter. Supplementary Fig. 2a shows that the $q = 1$ radius predicted by TRANSP-EFTP (which did not account for sawtooth activity) is much larger than the experimentally observed inversion radii R_{inv}. Our model profiles for $q(\overline{r})$ were chosen to match the TRANSP-EFTP profile only in the outer region of the plasma as shown in Supplementary Fig. 2b, whereas the inner portion shown in Supplementary Fig. 2c was modified within the limits of R_{inv} inferred from experiments. The MHD equilibria for our simulations were constructed using the code CHEASE [3], taking as input the modeled pressure profile in Supplementary Fig. 1a and the q profiles in Supplementary Fig. 2.

Besides being an essential physics ingredient for our study, the fairly flat q profile in Fig. 1c of the main article with $q \sim 1$ inside the $q = 1$ surface also has computational advantages. In combination with the suspected regularizing effect of MHD pressure mentioned in the previous paragraph, a flat q profile prevents the

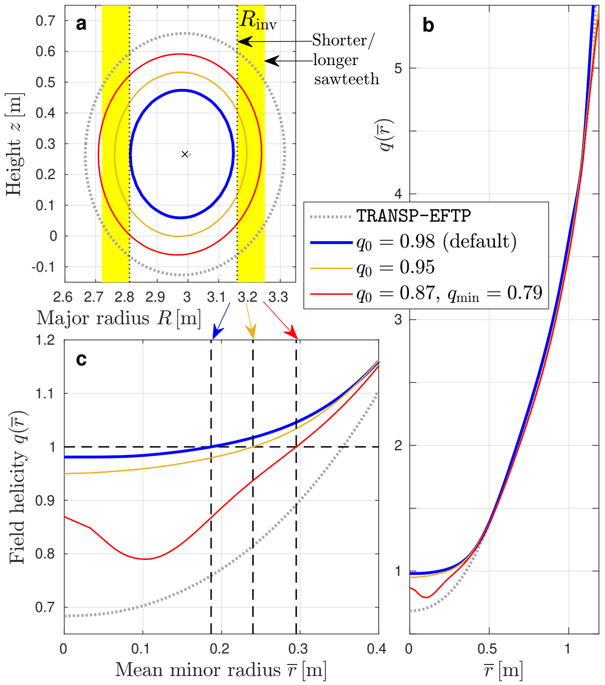

Supplementary Fig. 2. Modeling of the field helicity (safety factor) profile $q(\overline{r})$. Panel (**a**) shows the contours of the $q = 1$ radii in the poloidal (R, z) plane for the reference equilibrium computed by `TRANSP-EFTP` (dotted) and our three model profiles (solid). The yellow shaded areas indicate the approximate range of sawtooth inversion radii R_{inv} observed in the JET reference pulse 95679 that was used to constrain the $q = 1$ radii of the model profiles (before adjusting the pressure as shown in Supplementary Fig. 1). Panel (**b**) shows the full $q(\overline{r})$ profiles, whose structure in the central core is shown enlarged in (**c**).

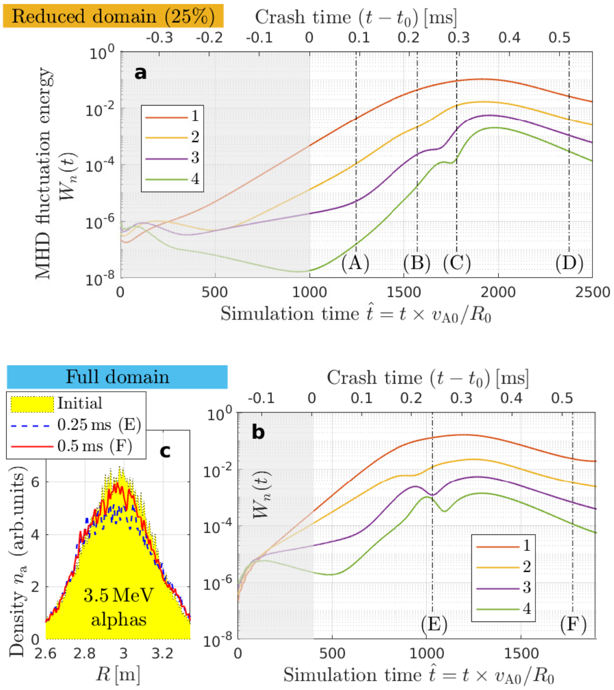

Supplementary Fig. 3. (**a**) Evolution of the four dominant toroidal harmonics $n = 1, 2, 3, 4$ of the fluctuating MHD field energy $W_n(t)$ in the case with $q_0 = 0.98$, simulating only the inner 25% of the magnetic flux space. This is a different view of the same instability as in Fig. 3a of the main article. (**b**) Evolution of $W_n(t)$ in the simulation of the full domain for the same case as in (**a**). (**c**) Evolution of the density profile $n_a(R)$ of 3.5 MeV alpha particles in the full-domain simulation, which confirms the result shown in Fig. 4e of the main article.

magnetic reconnection layer from collapsing rapidly into a narrow sheet, which would be difficult to resolve numerically even with the relatively small Lundquist and Reynolds numbers $S = \text{Re} = 10^6$ in our simulation. For instance, we were not able to perform a simulation using the steeply rising q profile of the original reference equilibrium from the `TRANSP-EFTP` simulation, which is shown as a dotted line in Supplementary Figs. 2b and 2c and has a low central value of $q_0 = 0.68$. A common feature of simulations that terminated abnormally is the formation of wide-spread magnetic chaos.

MHD simulations were successfully performed with the three model q profiles in Supplementary Fig. 2. The blue profile (default) with $q_0 = 0.98$ and $q = 1$ radius $\overline{r} \approx 0.19$ m has been studied most intensively, and the main results were reported in the main article. The other two profiles with $q_0 = 0.95$ at $\overline{r} \approx 0.24$ m (orange) and $q_0 = 0.97$ at $\overline{r} \approx 0.30$ m (red) were used for sensitivity tests, preliminary results of which are presented in Section below. Judging by the Alfvén mode activity seen in spectral analyses of experimental data [4], we speculate that our non-monotonic model q profile with $q_0 = 0.87$ and $q_{\min} = 0.79$ (red) may be closest to the conditions that were actually present during the quasi-steady stage in JET pulse 95679 that we used as a reference.

As is typical for simulations like ours, the MHD model includes a source term representing the tokamak's loop voltage, which balances the resistive dissipation of the initial current profile. Without this term, our central $q_0 = 0.98$ would rise above unity on the time scale of a few 100 μs, which implies that resistive dissipation participates in the simulated dynamics on a global scale. We emphasize again that simulating the collisionless reconnection process that is thought to occur during real Kadomtsev-type sawtooth crashes is still an active area of research [5]. MHD simulations like ours may thus be viewed as an attempt to mock up the micro-scale dynamics using resistive dissipation in such a way that the macro-scale dynamics are consistent with observations.

Supplementary Note 2. Fourier harmonics of the internal kink mode

With $N_\zeta = 96$ grid points along the toroidal angle ζ, our simulation can in principle capture toroidal Fourier harmonics $\exp(in\zeta)$ with toroidal mode num-

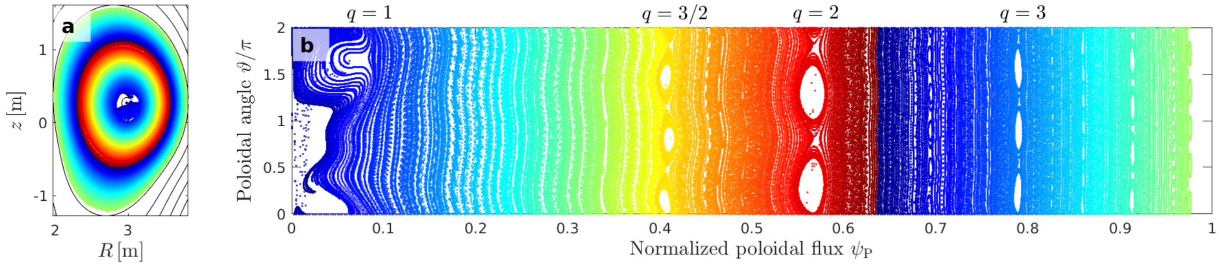

Supplementary Fig. 4. Poincaré plots of the magnetic topology after the sawtooth crash in the full-domain simulation in Supplementary Fig. 3b for snapshot (E) at 0.25 ms. Magnetic flux surfaces can be discerned by their colors. For orientation, panel (**a**) shows the result in cylinder coordinates (R, z) at $\zeta_0 = 0$. Panel (**b**) shows the same data in polar coordinates $(\psi_{\rm P}, \vartheta)$, where magnetic islands can be clearly seen. The normalized poloidal flux $0 \leq \psi_{\rm P} \leq 1$ is used as a minor radial coordinate ($0 =$ axis, $1 =$ edge) and is approximately proportional to $\overline{r}^2$ [1]. The locations of resonant surfaces with $q = 1$, 3/2, 2/1, 3/1 are indicated.

bers $-48 \leq n \leq 48$. Our simulation starts from a small initial perturbation applied to the Fourier harmonics $n = 1, 2, 3, 4$. Their evolution is shown in Supplementary Fig. 3 in terms of the MHD fluctuation energy $W_n(t)$ as defined in Eq. (18) of Ref. [6]. After the initial mode structure formation process, the internal kink instability in this simulation is clearly dominated by the $n = 1$ harmonic at all times.

Most results reported in the main article were obtained by simulating only the inner 25% of the poloidal magnetic flux space and placing a non-slip boundary along the red line in Fig. 1 of the main article. The evolution of the MHD fluctuation energy in that case is shown in Supplementary Fig. 3a. That simulation ran for a few 100 μs before macroscopic transport became visible, and we measure the crash time $t - t_0$ starting approximately from that instant. Here, we have chosen $t_0 = 0.38$ ms, which corresponds to $\hat{t}_0 = t_0/\tau_{\rm A0} = 1000$ in units of the toroidal Alfvén time $\tau_{\rm A0} = R_0/v_{\rm A0}$. Supplementary Fig. 3a shows that the dominant harmonics are still growing exponentially at that time.

Supplementary Note 3. Comparison with full-domain simulation: Mode evolution, fast alphas & peripheral islands

Simulating only the reduced domain facilitated parameter scans and convergence tests by reducing the computational effort. However, the artificial non-slip boundary located at the red line in Fig. 1 of the main article has a stabilizing effect on the internal kink instability. Moreover, it enhances the prompt loss of a certain group of fast alpha particles that are subject to large magnetic drifts [1]. Therefore, we performed some our our simulation in the full domain.

A comparison between Supplementary Fig. 3a and 3b shows that the MHD fluctuations grow more rapidly in the full domain. Nevertheless, the sawtooth crash still has the same overall time scale of a few 100 μs and the $n = 1$ harmonic is dominant in both simulations.

Supplementary Fig. 3c shows the density profile of 3.5 MeV alphas before, during and after the crash. Apart from the larger noise (which is due to the smaller number of particles per grid cell), the result is very similar to that shown in Fig. 4e of the main article for the reduced domain. Since the form of the velocity distribution is affected by the additional boundary losses in the reduced domain, our analyses of the alpha particle redistribution in pitch angle and energy were performed using the results of full-domain simulations (see Fig. 5 of the main article, and Supplementary Figs. 7 and 8 below).

Returning once more to the stabilizing effect of the artificial non-slip boundary: this effect can be understood by noting that the magnetic flux surfaces across the entire plasma radius are distorted in the presence of an internal kink mode. A larger amount of work is required to deform magnetic flux surfaces when the rigid boundary is closer to the $q = 1$ surface, so the kink mode's growth rate is smaller in simulations with a smaller plasma.

Conversely, in the full domain, the kink can cause distortions of other low-rational resonant surfaces, such as $q = 3/2$, 2/1, 3/1, ..., and lead to the formation of magnetic islands (or enlarge existing ones) through driven magnetic reconnection. This is an actual problem in real plasmas that affects their performance and may lead to disruptions that terminate the discharge. For this reason, giant sawtooth crashes should be avoided. This was, in fact, one motivation for our interest in cases with $q \sim 1$.

Nevertheless, even the benign sawtooth crash in our simulations with $q_0 = 0.98$ does cause magnetic reconnection at other resonant surfaces as shown in Supplementary Fig. 4. These islands are however sufficiently small and sufficiently far apart to avoid overlaps, the spread of chaos and associated loss of confinement. In any case, it must be kept in mind that Supplementary Fig. 4 is the result of a resistive MHD simulation with $S = 10^6$, which may not be representative for the situation in a real and effectively collisionless tokamak plasma. The islands in Supplementary Fig. 4 may be larger than they would be in a real plasma with JET parameters.

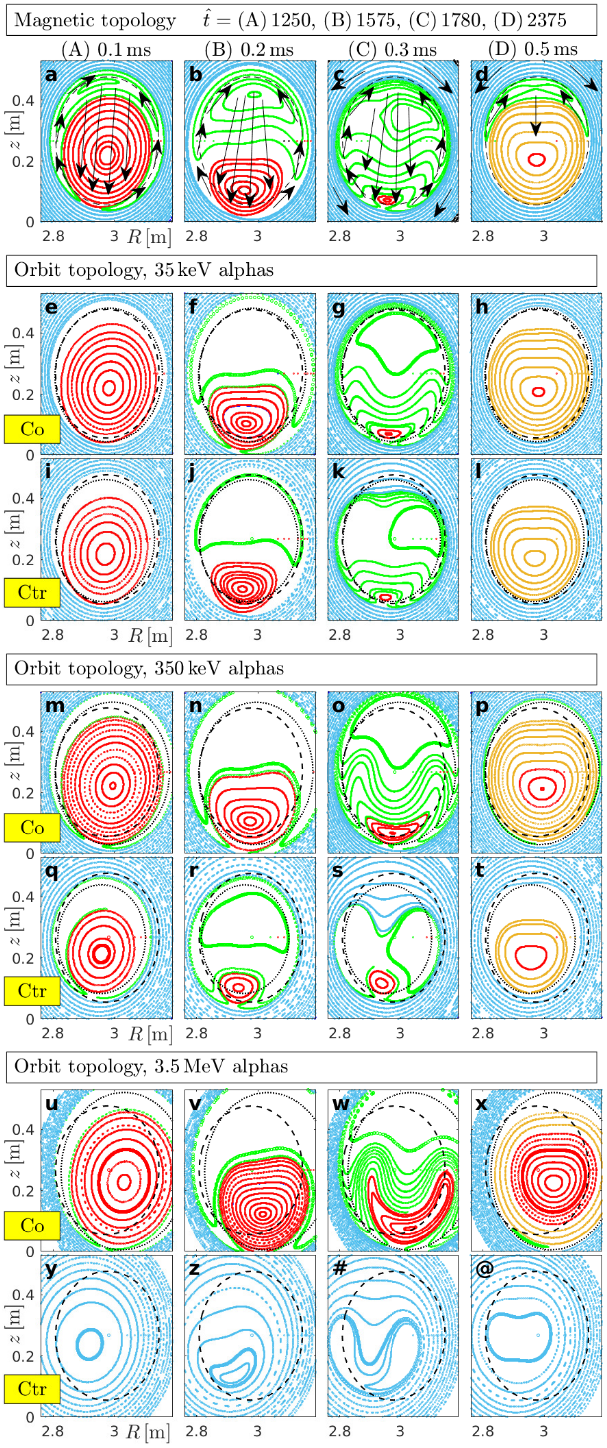

Supplementary Fig. 5. Topology of the magnetic field (**a**-**d**) and alpha particle orbits (**e**-**z**,#,**@**) at the times of snapshots (A)–(D) indicated in Supplementary Fig. 3 (and in Fig. 3a of the main article). Red and orange Poincaré contours have helicities $q, h < 1$. Reconnected green islands and unreconnected blue periphery have $q, h > 1$. The initial $q = 1$ surface is drawn as a dashed circle and the dotted black circles represent the initial $h = 1$ surfaces in cases where they exist (co-passing). Arrows roughly indicate the direction of electric drifts. Note that the density of the field contours and orbit contours in these plots is arbitrary, so it does not by any means reflect the density of magnetic flux or orbit surfaces, especially in the perturbed domain.

Supplementary Note 4. Guiding center orbit topology

The Poincaré plots for the magnetic field shown in Fig. 3b-d of the main article were computed by following test particles without magnetic drifts. These plots are shown again in the top row of Supplementary Fig. 5, with the addition of a fourth snapshot (D) taken near the end of the simulation at 0.5 ms.

The rest of Supplementary Fig. 5 shows the Poincaré contours of co- and counter-passing alpha particles with energies 35 keV, 350 keV and 3.5 MeV. Supplementary Figs. 5w and 5# appeared in box (ii) of Fig. 6 of the main article to illustrate that magnetic drifts reduce the amount of reconnection that occurs in orbit topology. The series of snapshots in Supplementary Fig. 5 show how the topology changes in time during the sawtooth crash and how the influence of the magnetic drifts increases with increasing particle energy.

The orange contours appearing in snapshot (D) in the right column of Supplementary Fig. 5 are meant to represent regions where $q < 1$ has been restored due to the effect of the loop voltage (current source), which balances the global dissipation of the plasma current by the relatively large resistivity (small Lundquist number $S = 10^6$) in our simulations. The source term gradually restores the initial q profile and would later lead to the growth of a new instability. However, the effect of the current source on the Poincaré plots is difficult to quantify, so the boundary between red and orange domains was chosen somewhat arbitrarily, based on no more than an intuitive guess.

It is interesting to note that all Poincaré plots for our default case with $q_0 = 0.98$ consist of closed contours. No chaotic domains were seen in this case, which means that there are no significant resonance overlaps. The same is true for the case with $q_0 = 0.95$, whereas signatures of chaos near the mixing radius can be seen in the non-monotonic case with $q_0 = 0.87$ that will be discussed in Section below (Supplementary Fig. 11).

Our Poincaré analyses were focused on particles in the deeply passing domain; that is, on pitch angles near ±90 degrees: $v_\parallel/v = \sin(\pm 0.48\pi) \approx \pm 1$. For completeness, Supplementary Fig. 6 shows the pitch dependence of the $h = 1$ resonance radius for alpha particles with kinetic energy $K = 3.5$ MeV. Both the inner (high-field-side) and outer (low-field-side) radius is shown. The respective points closest to the magnetic axis — namely, $X_{\rm HFS} = -0.154$ m, $\bar{r}_{\rm HFS} = 0.168$ m and $X_{\rm LFS} = 0.255$ m, $\bar{r}_{\rm LFS} = 0.288$ m — correspond to the points where the red curve in the main article's Fig. 7 crosses 1. One can see in Supplementary Fig. 6 that the resonant radius quickly increases as the pitch is reduced towards the trapped-passing boundary, which is located near $\alpha \approx 0.15\pi$. This means that the internal kink mode can be expected to have a smaller influence on particles with smaller pitch angles, which is consistent with our simulation results in Figs. 5e and 5f of the main article. Mirror-trapped

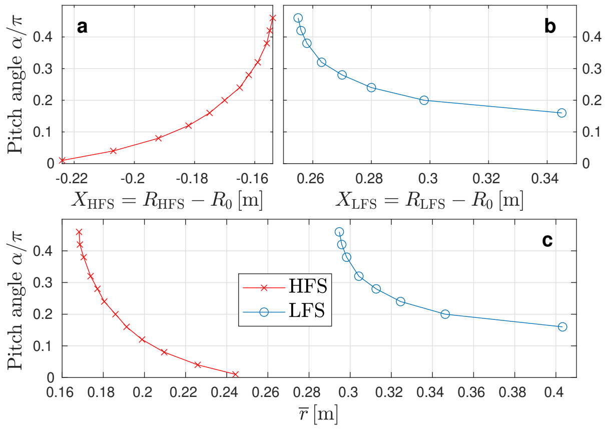

Supplementary Fig. 6. High-field-side (HFS, $R < R_0$) and low-field-side (LFS, $R > R_0$) radii of the $h = 1$ resonance on the midplane ($z_0 = 0.26$) for co-passing 3.5 MeV alpha orbits in the default case with $q_0 = 0.98$. The $h = 1$ resonance is absent for counter-passing alphas with 3.5 MeV in this case. The curves are plotted as functions of the radial distance from the plasma center at the midplane, using the major radial coordinate $X = R - R_0$ in (**a**,**b**), and the mean minor radius $\overline{r}$ in (**c**).

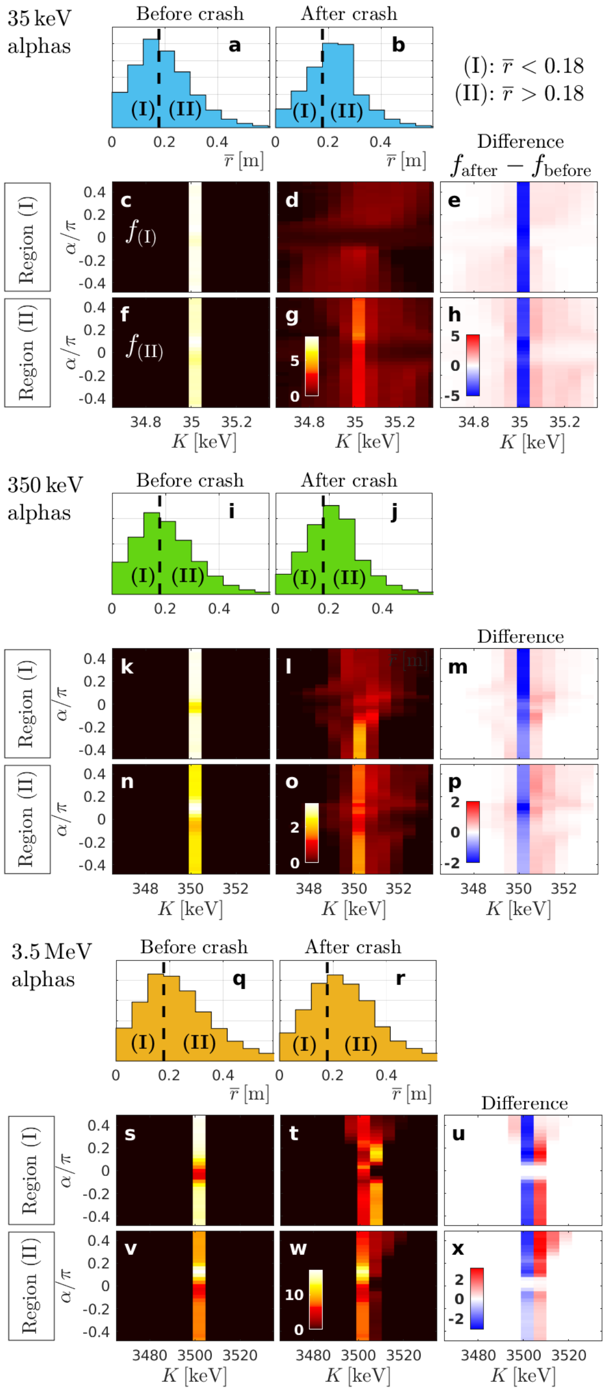

Supplementary Fig. 7. Evolution of the alpha particle velocity distribution with initial energies 35 keV (**a-h**), 350 keV (**i-p**), 3.5 MeV (**q-x**). The full domain is simulated in order to capture also large orbits at small values of α, which pass both through the plasma core and the periphery [1]. The left column shows the initial state before the crash, the central column shows the post-crash state, and the difference is shown on the right. Panels (**a**,**b**), (**i**,**j**) and (**q**,**r**) show histograms of particle numbers as functions of the mean minor radius $\overline{r}$. The vertical dashed line at $\overline{r} = 0.18$ m is near the $q = 1$ surface $\overline{r}_1$. The inner region $\overline{r} \lesssim \overline{r}_1$ and the outer region $\overline{r} \gtrsim \overline{r}_1$ are labeled (I) and (II). The pitch angle distributions for region (I) are shown in (**c-e**), (**k-m**) and (**s-u**), and those for region (II) are shown in (**f-h**), (**n-p**) and (**v-x**).

particles and counter-passing particles do not resonate at all with the internal kink in this configuration ($q_0 = 0.98$).

Supplementary Note 5. Particle redistribution in velocity space

In Fig. 5 of the main article, we showed how particles at different pitch angles are redistributed by the sawtooth crash in our default case with $q_0 = 0.98$. For that purpose, we measured the particle populations in an inner region $\overline{r} < 0.18$ m and an outer region $\overline{r} > 0.18$ m. Supplementary Fig. 7 provides a more detailed view of the data, showing also how the sawtooth affects the kinetic energy K of the particles, which had been initialized with a single value 35 keV (a-h), 350 keV (i-p) or 3.5 MeV (q-x).

In the region $0 \leq \overline{r} \leq 1.08$ m (or $0 \leq \overline{r}/\langle a \rangle \leq 0.9$ in normalized units), we divided the minor radial axis into 18 cells of size $\Delta\overline{r} = 0.06$ m. By counting the number of particles in each radial cell, before and after the sawtooth crash, we obtained the histograms shown in panels (a,b), (i,j) and (q,r) of Supplementary Fig. 7. We emphasize that these are histograms, not densities, so that the number of particles in each cell decreases towards the axis since the cell volume scales like $\Delta V \propto \overline{r} R \Delta\overline{r}$. The reason for using histograms instead of densities for the radial direction is that histograms show the amount of particle transport more clearly: the reduction seen in one region corresponds to the gain in another. This makes plots like those in Fig. 5 of the main article quantitatively intuitive.

For each cell $\overline{r}_i$ $(i = 1, ..., 18)$, we recorded the velocity distribution $f_i(K, \alpha)$ in the form of a true density function, which is a histogram divided by the velocity space Jacobian $\mathcal{J}_{K\alpha} \propto 2\pi v_\perp$ (cf. Eq. (A.12) of Ref. [1]). Their integration over an inner region (I) $\overline{r} < 0.18\,\mathrm{m}$ and an outer region (II) $\overline{r} > 0.18\,\mathrm{m}$ yielded the velocity distributions $f_{(\mathrm{I})}(K, \alpha)$ and $f_{(\mathrm{II})}(K, \alpha)$ as well as the difference $f_{\mathrm{after}} - f_{\mathrm{before}}$ shown in panels (c-h), (k-p) and (s-x) of Supplementary Fig. 7. Here, the density function $f(K, \alpha)$ was preferred over the histogram $H(K, \alpha) = \mathcal{J}_{K\alpha} f(K, \alpha)$ because an isotropic distribution becomes a constant along the pitch angle α when shown in terms of $f(K, \alpha)$ as in Supplementary Fig. 7c.

The boundary between regions (I) and (II) was put close to the initial $q = 1$ radius $\overline{r}_1 \approx 0.19\,\mathrm{m}$, since the latter corresponds roughly to the inversion radius of a sawtooth crash. Although neither the minor radius $\overline{r}$ nor the pitch angle α of an alpha particle is conserved (even during its unperturbed motion) in a tokamak, the resulting blurring of the measured distribution is acceptable at the level of detail that is of interest here, so we kept it simple and did not use orbit-based coordinates [7].

The main message that we wish to convey with Supplementary Fig. 7 is as follows. The majority of suprathermal alpha particles tend to be accelerated by the kink mode, which means that they have a stabilizing influence on the mode. Supplementary Fig. 7 shows this effect most clearly for the co-passing particles. Our simulations also reproduce the stabilizing influence of trapped particles [8–10], though this is not clearly visible in Supplementary Fig. 7 because the trapped particles in the domain $-0.15\pi \lesssim \alpha \lesssim 0.15\pi$ are spread out in energy so widely that many of them have left the narrow energy window plotted here and are no longer visible after the crash. (Their weights are however included in the energy-integrated plots of Fig. 5 of the main article.)

Some exceptions can also be seen and may be explained in terms of resonances. For instance, Supplementary Fig. 7u shows that some of the co-passing 3.5 MeV alphas around $\alpha \gtrsim +0.3\pi$ in region (I) are decelerated, which means that they drive the internal kink mode. This is consistent with the fact that we have initialized the simulation with a destabilizing density profile and that a significant portion of the $h = 1$ resonance of these particles lies within the $q = 1$ radius $\overline{r}_1 \approx 0.19$, as one can see from the values of $\overline{r}_{\mathrm{HFS}}$ in Supplementary Fig. 6c.

Supplementary Fig. 7e seems to indicate that slow alphas with 35 keV exert a net driving force in the counter-passing domain and a net damping force in the co-passing domain. However, the effect is subtle and one should exercise care when trying to interpret this observation because these particles are subject to violent transport: as we have seen in Fig. 4b of the main article, these particles move in and out and around the relaxation domain, so they may transiently resonate with the mode at one time or another whenever their orbit helicity matches that of the mode ($h = 1$). No attempt was made yet to untangle this process in detail.

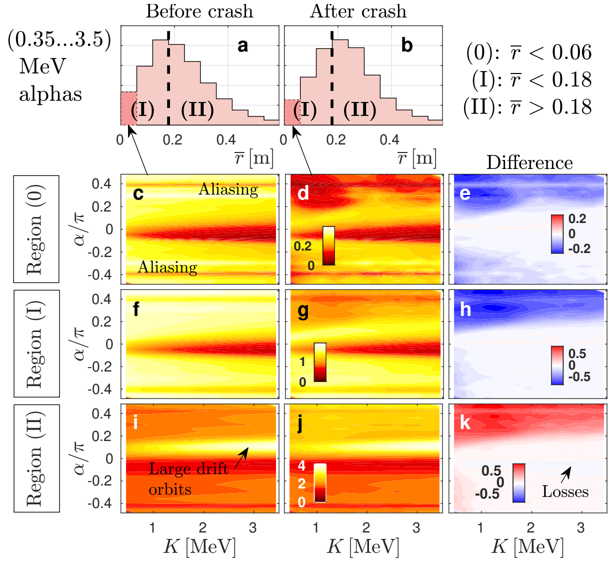

Supplementary Fig. 8. Evolution of the alpha particle velocity distribution in a simulation initialized with a uniform population of particles in the energy range $0.35\,\mathrm{MeV} \leq K \leq 3.5\,\mathrm{MeV}$ [1]. Panels (**a**,**b**,**f**-**k**) are made and arranged like Supplementary Fig. 7a-h. Added here is a third integration region $0 < \overline{r} < 0.06\,\mathrm{m}$ labeled 'Region (0)', results for which are shown in panels (**c**-**e**). Note that the horizontal stripes around $\alpha \approx \pm(0.3...0.4)\pi$ are aliasing artifacts that should be ignored (cf. Section 8.3 in Ref. [1]). This simulation was performed in the full domain, and only a vanishingly small amount of boundary losses is visible in (**k**).

Finally, it is interesting to inspect how smooth or sharp the energy threshold for the confinement of passing alpha particles is. For this purpose, Supplementary Fig. 8 shows the evolution of the velocity distribution $f(K, \alpha)$ in a simulation initialized with alpha particles distributed uniformly in the energy range $0.35\,\mathrm{MeV} \leq K \leq 3.5\,\mathrm{MeV}$.

In the domain of co-passing particles ($\alpha > 0$), the sawtooth-crash-induced transport can be seen to vary gradually. There is no sharp threshold in the energy window examined here. In the case of counter-passing particles ($\alpha < 0$), Supplementary Figs. 8h and 8k seem to indicate that there is a transport threshold near 0.5...1 MeV. However, as we have also noted in the main article, the observed amount of co-/counter-passing asymmetry is linked to the choice of the boundary between the inner and outer integration regions. To illustrate this, we have added in Supplementary Fig. 8c-e the results for a smaller inner region $\overline{r} < 0.06\,\mathrm{m}$ labeled 'Region (0)'. Supplementary Fig. 8e shows clearly that the transport of counter-passing alphas out of the small inner region (0) increases gradually when K is reduced from 3.5 MeV to sub-MeV values: transport begins to rise at large negative pitch angles ($\alpha \approx -\pi/2$) and then spreads towards the trapped-passing boundary ($\alpha \approx -0.15\pi$).

Supplementary Note 6. Gyroaveraging

As mentioned in the Methods section of the main article, gyroaveraging had no significant effect on the results of the simulations discussed in this work. Nevertheless, our simulations were performed using 4-point averaging over the Larmor radius as described in Ref. [6], and the associated smoothing effect contributes to the form of the density fields shown in Figs. 4 and 8 of the main article.

Supplementary Note 7. Parallel electric field

We note that another factor that may affect our artificial light particle species in Fig. 8a and 8b of the main article is the parallel electric field $E_\parallel = \mathbf{E}\cdot\mathbf{B}/B$ (here about 1% of $|\mathbf{E}_\perp|$) that is generated in the relaxing domain of our resistive MHD simulation. The larger charge-to-mass ratio Ze/M of the light particles makes them more responsive to $E_\parallel$ than alphas and heavier ions. The consequences of this remain to be examined.

Supplementary Note 8. Safety factor profile scan

The physical picture proposed in the main article implies that the selective confinement of fast alphas is sensitive with respect to the profile of the field helicity (= tokamak safety factor) $q(\overline{r})$. In order to highlight this effect, we present in this section preliminary results obtained from simulations with q profiles that have a central value q_0 lower than our default case with $q_0 = 0.98$. The two cases considered are shown in Supplementary Fig. 2: a monotonic q profile with $q_0 = 0.95$, and a non-monotonic q profile with $q_0 = 0.87$ and an off-axis minimum $q_{\rm min} = 0.79$ at $\overline{r} \approx 0.1\,{\rm m}$. Since the $q = 1$ radius is larger in these cases, we have increased the size of the simulation domain from 25% to 50% of the poloidal magnetic flux space.

The evolution of the MHD fluctuation energy in these two cases is shown in Supplementary Figs. 9a and 9c. One can see that the $n = 1$ harmonic dominates in both simulations. Comparison with Supplementary Figs. 3a and 3b shows that the growth rate of the internal kink in Supplementary Fig. 9 has increased with decreasing q_0, which — to be precise — is not primarily due to the value of q_0 itself but due to the steeper gradient that these $q(\overline{r})$ profiles have at the $q = 1$ radius, and which translates to a steeper gradient in the plasma current. This results in a higher $\mathbf{E}\times\mathbf{B}$ velocity and a shorter crash time as one can see in Supplementary Figs. 10a and 11a: the vertical displacement velocity v_{z0} at the magnetic axis reaches nearly $10\,{\rm km\,s^{-1}}$ in the case with $q_0 = 0.95$, and exceeds $20\,{\rm km\,s^{-1}}$ in the case with $q_0 = 0.87$. The implications for fast alpha confinement will be discussed shortly.

Before proceeding, we would like to insert a note of caution. Although low central values of the field helicity

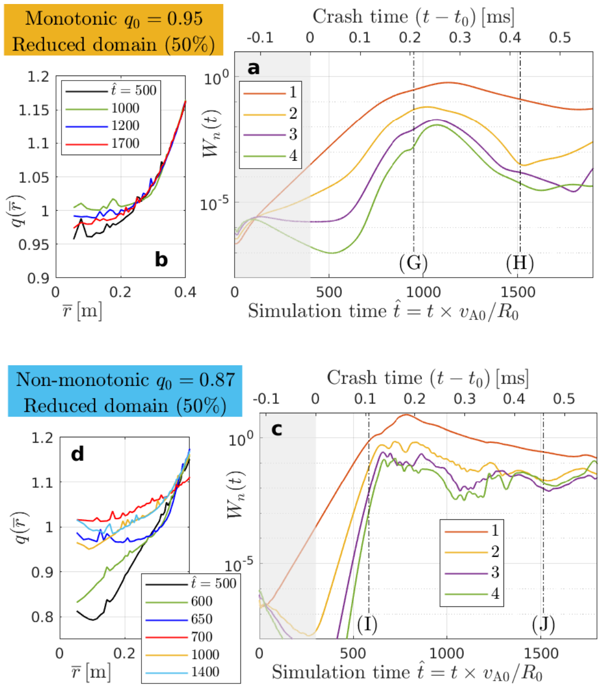

Supplementary Fig. 9. Comparison of the evolution of (**a**,**c**) the MHD fluctuation energy $W_n(t)$ and (**b**,**d**) the q profile in the cases initialized with $q_0 = 0.95$ and $q_0 = 0.87$ (cf. Supplementary Fig. 2). Panels (**a**) and (**c**) are arranged like Supplementary Figs. 3a and 3b. The labels (G)-(J) indicate the snapshot times for which alpha particle density profiles are plotted in Supplementary Figs. 13 and 14 below. The q profiles in (**b**,**d**) represent field helicities averaged over the initial (unperturbed) magnetic surfaces.

like $q_0 \sim 0.87$ in our third model scenario are consistent with some experimental observations, this parameter alone does not determine the sawtooth crash dynamics. For instance, the crash can be expected to proceed more slowly when the q profile is initialized with a flat shoulder around the $q = 1$ radius [11, 12]. This is one of the reasons why the present safety factor scan has only a preliminary character: the exact shape of the profile matters, so the cases we have simulated and discuss here should be viewed as mere random examples.

Supplementary Figs. 9b and 9d show the evolution of q profiles in the cases with $q_0 = 0.95$ and 0.87. These plots show how the central portion of the q profile tends to rise above unity during the crash phase and then oscillates within a few percent around $q \sim 1$ until the $\mathbf{E}\times\mathbf{B}$ flow has decayed to an insignificant level (and sources begin to restore the initial profile). These perturbed profiles must also be interpreted with care because we have simply averaged the field helicity over the *unperturbed* flux surfaces (each identified by a unique value of $\overline{r}$) of the initial MHD equilibrium. Ideally, the perturbed field helicity profiles should be computed in accordance with the perturbed magnetic surfaces in order to be meaningful. Since this was not done here, the plots in Supplementary Figs. 9b and 9d are meant to show only the overall trend.

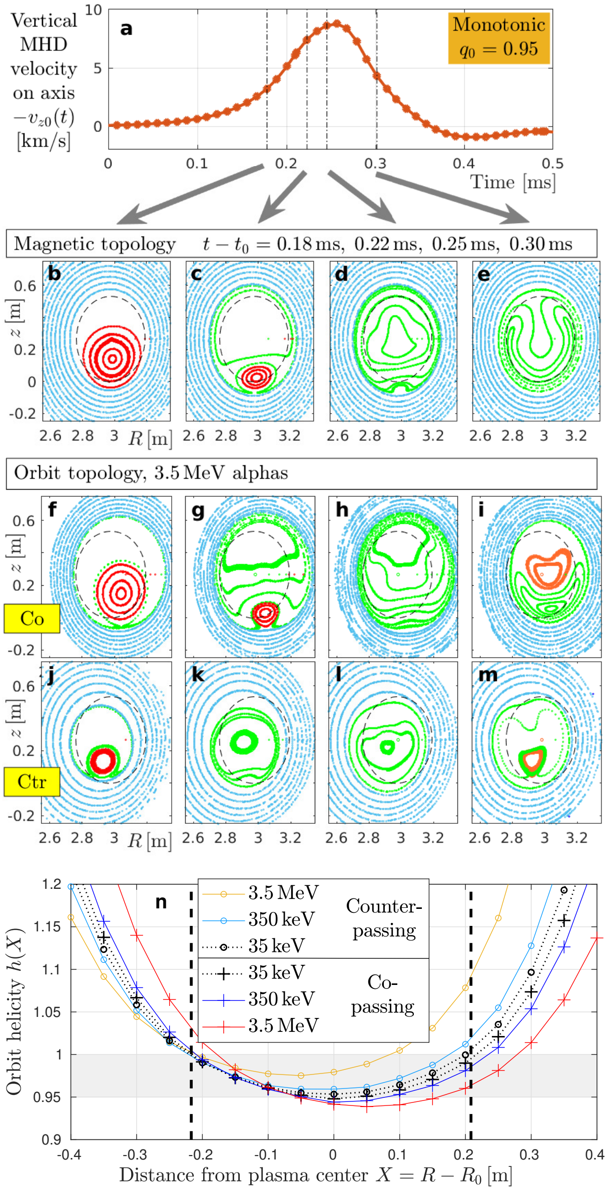

Supplementary Fig. 10. Overview of the sawtooth crash dynamics in the case with monotonic q profile and central value $q_0 = 0.95$. (a) Evolution of the vertical MHD velocity $v_{z0}(t)$ at the magnetic axis as in Fig. 3a of the main article. Vertical dash-dotted lines mark the times for which Poincaré plots are shown in the panels below (arrows). (**b-m**) Poincaré plots showing the topology of the magnetic field and alpha particle orbits with energy $K = 3.5$ MeV and pitch angles $\alpha = \pm 0.48\pi$. Arranged similarly to Supplementary Fig. 5. (**n**) Orbit helicity profiles $h(X)$, arranged like in Fig. 7 of the main article.

The actual evolution of the magnetic topology can be seen in Supplementary Figs. 10b-e and 11b-e. Both cases exhibit a Kadomtsev-type crash followed by Wesson-type overshoots of the $\mathbf{E} \times \mathbf{B}$ flow. At later times, Supplementary Figs. 10a and 11a show a temporary reversal of the flow direction at the original location of the magnetic axis, which seems to be a signature of increasingly turbulent motion (perhaps the kink's return flows become Kelvin-Helmholtz unstable). Note that the volume integrated MHD fluctuation energy in Supplementary Fig. 9 is still large at that time.

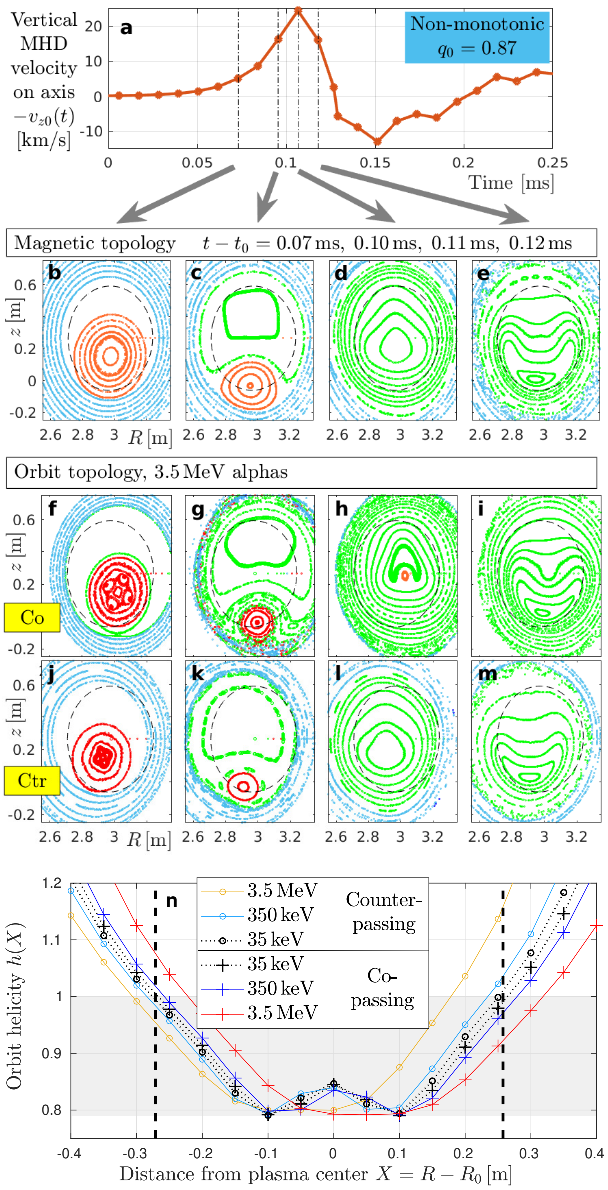

Supplementary Fig. 11. Overview of the sawtooth crash dynamics in the case with non-monotonic q profile and central value $q_0 = 0.87$. All panels (**a-n**) are made and arranged like those in Supplementary Fig. 10.

The Poincaré plots of the orbit topology show that there are still readily visible differences between co- and counter-passing 3.5 MeV alphas in the case with $q_0 = 0.95$ in Supplementary Fig. 10, while the overall structures begin to look similar in the case with $q_0 = 0.87$ in Supplementary Fig. 11. This, of course, can be expected from the fact that the magnitude of the magnetic drifts remains more or less the same, while the $q = 1$ radius increases in our two model profiles.

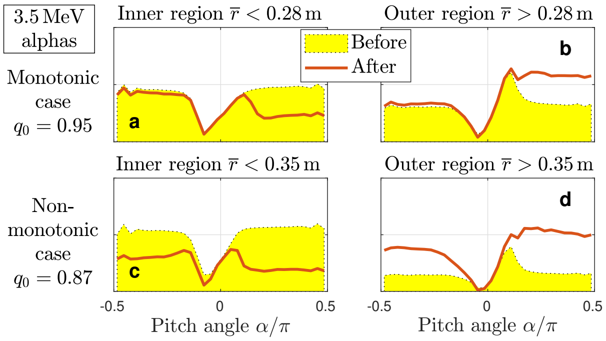

Supplementary Fig. 12. Pre- and post-crash distributions in pitch angle $\alpha = \sin^{-1}(v_\parallel/v)$ for 3.5 MeV alphas in the cases with $q_0 = 0.95$, $\bar{r}_1 = 0.24$ m (**a**,**b**) and $q_0 = 0.87$, $\bar{r}_1 = 0.3$ m (**c**,**d**). The boundary between integration regions (I) and (II) is chosen to be somewhat larger than the respective $q = 1$ radius $\bar{r}_1$. Arranged like Fig. 5 of the main article.

In contrast to our default case in Fig. 7 of the main article, the orbit pitch profiles in Supplementary Figs. 10n and 11n all lie below unity in the plasma center, so there are $h = 1$ resonances and hence reconnection occurs in the orbit topology of both co- and counter passing 3.5 MeV alphas. However, in the case with $q_0 = 0.95$, the $h = 1$ resonance for counter-passing 3, 5 MeV alphas lies almost entirely within the $q = 1$ radius $\bar{r}_1 \approx 0.24$ m, so that these particles tend to be mixed only inside that region. This can be verified in Supplementary Figs. 12a and 12b, which show that effectively no transport occurs at negative pitch angles $\alpha < 0$ across the radius $\bar{r} = 0.28$ m that we have chosen as a boundary between the inner region (I) and the outer region (II). The situation is different in the case with $q_0 = 0.87$, where a part of the $h = 1$ resonance lies outside the $q = 1$ radius $\bar{r}_1 \approx 0.3$ m and where the mixing radius extends far beyond the initial $q = 1$ radius due to the larger amount of reconnected magnetic flux (e.g., see Fig. 8.12 of [13]). For this case, Supplementary Figs. 12c and 12d show that there is significant transport of both co- and counter-passing 3.5 MeV alphas across the chosen boundary $\bar{r} = 0.35$ m between region (I) and (II).

Another notable and interesting difference between the two cases that can be seen in the Poincaré plots in Supplementary Figs. 10 and 11 is that the field and orbit topology in the monotonic case with $q_0 = 0.95$ still consists of good Kolmogorov-Arnold-Moser (KAM) surfaces on global scales, while the non-monotonic case with $q_0 = 0.87$ exhibits signatures of chaos near the boundary of the $q \sim 1$ (Supplementary Fig. 11e) and $h \sim 1$ domain (Supplementary Figs. 11g, 11k and onward). Such behavior is an indication of the existence of multiple resonances that have different helicities and coexist in the same domain (= nonlinear resonance overlap).

While the consequences of resonance overlaps are understood, the reason for why multiple resonances coexist in our non-monotonic case with $q_0 = 0.87$ but not in the other cases remains to be clarified. We note that, during the early stages of the sawtooth crash, the non-monotonic case has a resonance with $h = 4/5 > q_{\rm min} = 0.79$. Having a poloidal peridicity of 4, this resonance is likely to be the reason for the island chain that can be seen near the orbit axis in Supplementary Figs. 11f and 11j. Similar structures were reported in Ref. [14]. Indeed, at the time of this snapshot ($t - t_0 \approx 0.07$ ms, $\hat{t} \approx 500$) the minimum of the q profile in Supplementary Fig. 9d is still below 0.8. Shortly thereafter (not shown), we observe an island chain with poloidal periodicity of 5 in the co-passing orbits, which is indicative of an $h = 5/6 = 0.8\overline{3}$ resonance in close proximity of the reconnection layer. The topological distortions associated with these islands may also couple to the above-mentioned modulations of MHD flows (possibly leading to MHD turbulence). The causality (if any) of the underlying processes is unclear. One phenomenon may be the trigger of the other, or they may both be manifestations of one secondary nonlinear instability. Whatever is happening, it leads here to the formation of a chaotic belt, especially in the case of co-passing particles in Supplementary Fig. 11g.

Finally, we inspect the spatial transport of monoenergetic alpha particles with $K = 35$ keV, 350 keV and 3.5 MeV. The results are summarized in Supplementary Figs. 13 and 14. In both cases, fast alphas with 3.5 MeV are still better confined than slow alphas with 35 keV. However, in comparison with our default case with $q_0 = 0.98$ in Fig. 4e of the main article, the reduction of the fast alpha density in the plasma center has become significant: a drop by about 30% and 60% is seen in Supplementary Fig. 13e and 14e, respectively. Obviously, such kinds of sawtooth crashes are less attractive for the control of helium ash than our default case in Fig. 4 of the main article.

The trend is consistent with the underlying physical picture that we have described in the main article. We have already seen in Supplementary Figs. 10n and 11n that the $h = 1$ resonances exist also for counter-passing particles in these cases, so that their orbit topology is subject to reconnection. In other words, by reducing q_0, we have lost much of the orbit topology's sensitivity with respect to magnetic drifts that was shown in box (ii) of Fig. 6 of the main article.

More detailed analysis is required to evaluate the ratio $\tau_{2\pi}/\tau_{\rm crash}$ of resonance detuning and sawtooth crash times, whose role was illustrated in boxes (iii) and (iv) of Fig. 6 of the main article. A first rough inspection indicates that this part of the synergism is also broken. We have already seen in Supplementary Figs. 10 and 11 that the crash times $\tau_{\rm crash}$ have become shorter by about a factor 2–4. This may be partly compensated by the larger pre-crash values of the parameter $|1 - h|$, which would imply a correspondingly shorter detuning time $\tau_{2\pi}$. However, the perturbed q profiles in Supplementary Figs. 9b and 9d approach unity at the time when the $\mathbf{E} \times \mathbf{B}$ velocity peaks, so the detuning time $\tau_{2\pi}$ may in fact be as long as in the $q_0 = 0.98$ case, implying that even fast alphas are unable to detach from the kink due to the shorter crash time $\tau_{\rm crash}$.

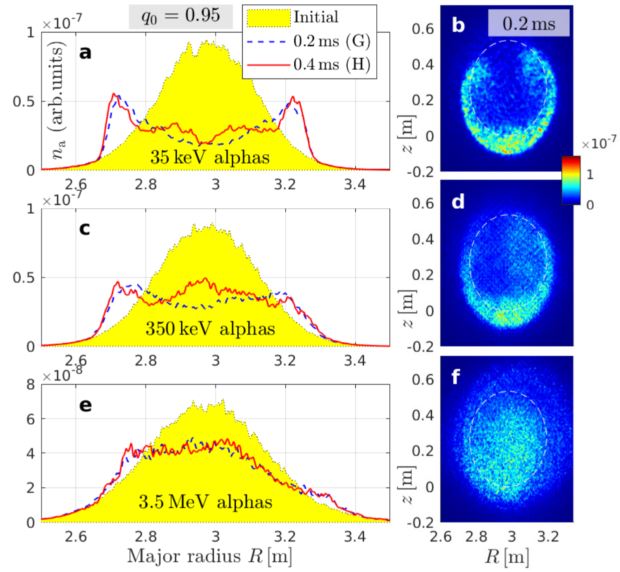

Supplementary Fig. 13. Spatial transport of alpha particles with kinetic energies $K = 35\,\text{keV}$ (**a**,**b**), 350 keV (**c**,**d**), 3.5 MeV (**e**,**f**) during the sawtooth crash in Supplementary Fig. 10 in the case with $q_0 = 0.95$. The snapshot times (G) and (H) were indicated in Supplementary Fig. 9a. Arranged like Fig. 4 of the main article, but showing a larger spatial domain. These data were obtained in simulations of a reduced domain covering the inner 50% of the poloidal magnetic flux space.

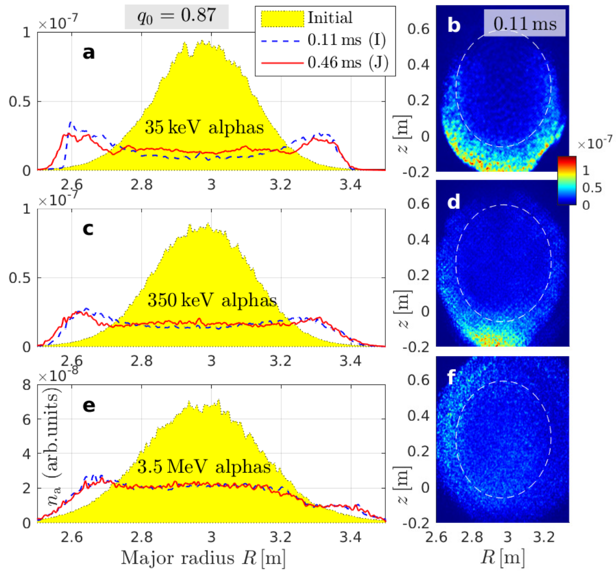

Supplementary Fig. 14. Spatial transport of alpha particles with kinetic energies $K = 35\,\text{keV}$ (**a**,**b**), 350 keV (**c**,**d**), 3.5 MeV (**e**,**f**) during the sawtooth crash in Supplementary Fig. 11 in the case with $q_0 = 0.87$. The snapshot times (I) and (J) were indicated in Supplementary Fig. 9c. Arranged like Fig. 4 of the main article, but showing a larger spatial domain. These data were obtained in simulations of a reduced domain covering the inner 50% of the poloidal magnetic flux space.

Evidence for this can be seen in the redistribution of 350 keV alphas in Supplementary Fig. 13d for $q_0 = 0.95$, which develop the same horseshoe-like structure as the 35 keV alphas in the main article's Fig. 4b for $q_0 = 0.98$. This implies that the energy threshold beyond which alpha particles can decouple from the kink has increased substantially, possibly even above the energy of newly-born 3.5 MeV alphas.

In the case with $q_0 = 0.87$, off-axis humps can be seen in the post-crash density profiles even for 3.5 MeV alphas, which implies that their redistribution has become akin to that of an MHD fluid. A theory for this regime was proposed by Kolesnichenko *et al.* [12], and this has been suggested as an explanation for observed redistribution of alpha particles in the intermediate energy range 150...600 keV in TFTR experiments [15]. Although the measurements in the plasma center suffered from relatively large uncertainties [16], our simulation results in Supplementary Figs. 13 and 14 support this possibility in principle.

Note that the 3.5 MeV alpha particle density profile in Supplementary Fig. 14e has developed a hump on the high-field side ($R \approx 2.7\,\text{m}$) and a shoulder on the low-field side ($R \approx 3.3\,\text{m}$). This is most likely a manifestation of the different redistribution of co- and counter-passing alpha profiles, showing that magnetic drifts have clearly visible effects even in the case initialized with low $q_0 = 0.87$.

Supplementary References

[1] A. Bierwage, M. Fitzgerald, Ph. Lauber, M. Salewski, Y. Kazakov, and Ž. Štancar. Representation and modeling of charged particle distributions in tokamaks. *Comp. Phys. Comm.*, 275:108305, 2022.

[2] Ž. Štancar, Z. Ghani, J. Eriksson, A. Žohar, S. Conroy, Ye. O. Kazakov, T. Craciunescu, M. Nocente, L. Garzotti, V. Radulović, P. Sirén, V. Kiptily, K. Kirov, Y. Baranov, G. Szepesi, M. Dreval, M. Gorelenkova, H. Weisen, E. Militello-Asp, L. Snoj, and JET Contributors. Experimental validation of an integrated modelling approach to neutron emission studies at JET. *Nucl. Fusion*, 61:126030, 2021.

[3] H. Lütjens, A. Bondeson, and O. Sauter. The CHEASE code for toroidal MHD equilibria. *Comp. Phys. Comm.*, 97:219, 1996.

[4] Ye. O. Kazakov, J. Ongena, J.C. Wright, S.J. Wukitch, V. Bobkov, J. Garcia, V.G. Kiptily, M. J. Mantsinen, M. Nocente, M. Schneider, H. Weisen, Y. Baranov, M. Baruzzo, R. Bilato, A. Chomiczewska, R. Coelho, T. Craciunescu, K. Cromb, M. Dreval, R. Dumont, P. Dumortier, F. Durodié, J. Eriksson, M. Fitzgerald, J. Galdon-Quiroga, D. Gallart, M. Garcia-Mu noz, L. Giacomelli, C. Giroud, J. Gonzalez-Martin, A. Hakola, P. Jacquet, T. Johnson, A. Kappatou, D. Keeling, D. King, K.K. Kirov, P. Lamalle, M. Lennholm, E. Lerche, M. Maslov, S. Mazzi, S. Menmuir, I. Monakhov, F. Nabais, M.F.F. Nave amd R. Ochoukov, A.R. Polevoi, S.D. Pinches, U. Plank, D. Rigamonti, M. Salewski, P.A. Schneider, S.E. Sharapov, Ž. Štan-

car, A. Thorman, D. Valcarcel, D. Van Eester, M. Van Schoor, J. Varje, M. Weiland, Z.N. Wendler, JET Contributors, ASDEX Upgrade Team, EUROfusion MST1 Team, and Alcator C-Mod Team. Physics and applications of three-ion ICRF scenarios for fusion research. *Phys. Plasmas*, 28(2):020501, 2021.

[5] R. Kumar, A. Bhattacharjee, and F. Ebrahimi. Kinetic simulations of the sawtooth crash. In *Bulletin of the 63rd APS DPP, 2021, Pittsburgh, PA, USA*. APS, 2021. https://meetings.aps.org/Meeting/DPP21/Session/GP11.30.

[6] A. Bierwage, Y. Todo, N. Aiba, and K. Shinohara. Sensitivity study for N-NB-driven modes in JT-60U: Boundary, diffusion, gyroaverage, compressibility. *Nucl. Fusion*, 56(10):106009, 2016.

[7] A. Bierwage and K. Shinohara. Orbit-based analysis of resonant excitations of Alfvén waves in tokamaks. *Phys. Plasmas*, 21(11):112116, 2014.

[8] F. Porcelli. Fast particle stabilisation. *Plasma Phys. Control. Fusion*, 33(13):1601, 1991.

[9] F. Porcelli, R. Stankiewicz, H.L. Berk, and Y.Z. Zhang. Internal kink stabilization by high-energy ions with non-standard orbits. *Phys. Fluids B*, 4(10):3017, 1992.

[10] F. Porcelli, R. Stankiewicz, W. Kerner, and H.L. Berk. Solution of the drift-kinetic equation for global plasma modes and finite particle orbit widths. *Phys. Plasmas*, 1(3):470, 1994.

[11] H. Soltwisch, W. Stodiek, J. Manickam, and J. Schliiter. ... In *Plasma Physics and Controlled Nuclear Fusion Research 1986*, volume 1, page 433. IAEA, Vienna, 1987.

[12] Ya.I. Kolesnichenko, Yu.V. Yakovenko, D. Anderson, M. Lisak, and F. Wising. Sawtooth oscillations with the central safety factor, q_0, below unity. *Phys. Rev. Lett.*, 68(26):065001, 1992.

[13] R. B. White. *The Theory of Toroidally Confined Plasmas*. Imperial College Press, London, 3rd edition, 2014.

[14] Ya.I. Kolesnichenko, V.V. Lutsenko, R.B. White, and Yu.V. Yakovenko. Theory of resonance influence of sawtooth crashes on ions with large orbit width. *Phys. Plasmas*, 5(8):2963, 1998.

[15] B.C. Stratton, R.J. Fonck, G.R. McKee, R.V. Budny, Z. Chang, and F. Wising. Observation of sawtooth redistribution of non-thermal, confined alpha particles in TFTR DT discharges. *Nucl. Fusion*, 36(11):1586, 1996.

[16] G.R. McKee, R.J. Fonck, B.C. Stratton, R.V. Budny, Z. Chang, and A.T. Ramsey. Transport measurements for confined non-thermal alpha particles in TFTR DT plasmas. *Nucl. Fusion*, 37(4):501, 1997.

JET Contributors

N. Abid[5], K. Abraham[5], P. Abreu[11], O. Adabonyan[5], P. Adrich[12], M. Afzal[5], T. Ahlgren[13], L. Aho-Mantila[14], N. Aiba[2], M. Airila[14], M. Akhtar[5], R. Albanese[16], M. Alderson-Martin[5], D. Alegre[17], S. Aleiferis[18], A. Aleksa[5], E. Alessi[8], P. Aleynikov[19], J. Algualcil[20], M. Ali[5], M. Allinson[5], B. Alper[5], E. Alves[11], G. Ambrosino[16], R. Ambrosino[16], E. Andersson Sundén[22], P. Andrew[19], B.M. Angelini[21], C. Angioni[6], I. Antoniou[5], L.C. Appel[5], C. Appelbee[5], S. Aria[5], M. Ariola[16], G. Artaserse[21], W. Arter[5], V. Artigues[6], N. Asakura[1,2], A. Ash[5], N. Ashikawa[23], V. Aslanyan[24], M. Astrain[25], O. Asztalos[26], D. Auld[5], F. Auriemma[27], Y. Austin[5], L. Avotina[28], E. Aymerich[29], A. Baciero[17], F. Bairaktaris[30], J. Balbin[10], L. Balbinot[27], I. Balboa[5], M. Balden[6], C. Balshaw[5], N. Balshaw[5], V.K. Bandaru[6], J. Banks[5], Yu.F. Baranov[5], C. Barcellona[31], A. Barnard[5], M. Barnard[5], R. Barnsley[19], A. Barth[5], M. Baruzzo[21], S. Barwell[5], M. Bassan[19], A. Batista[11], P. Batistoni[21], L. Baumane[28], B. Bauvir[19], L. Baylor[32], P.S. Beaumont[5], D. Beckett[5], A. Begolli[5], M. Beidler[32], N. Bekris[33,34], M. Beldishevski[5], E. Belli[35], F. Belli[21], É. Belonohy[5], M. Ben Yaala[36], J. Benayas[5], J. Bentley[5], H. Bergsåker[37], J. Bernardo[11], M. Bernert[6], M. Berry[5], L. Bertalot[19], H. Betar[38], M. Beurskens[39], S. Bickerton[5], B. Bieg[40], J. Bielecki[41], A. Bierwage[1,2], T. Biewer[32], R. Bilato[6], P. Bílková[42], G. Birkenmeier[6], H. Bishop[5], J.P.S. Bizarro[11], J. Blackburn[5], P. Blanchard[43], P. Blatchford[5], V. Bobkov[6], A. Boboc[5], P. Bohm[42], T. Bohm[44], I. Bolshakova[45], T. Bolzonella[27], N. Bonanomi[6], D. Bonfiglio[27], X. Bonnin[19], P. Bonofiglo[46], S. Boocock[5], A. Booth[5], J. Booth[5], D. Borba[11,33], D. Borodin[47], I. Borodkina[42,47], C. Boulbe[48], C. Bourdelle[10], M. Bowden[5], K. Boyd[5], I. Božičević Mihalić[49], S.C. Bradnam[5], V. Braic[50], L. Brandt[51], R. Bravanec[52], B. Breizman[53], A. Brett[5], S. Brezinsek[47], M. Brix[5], K. Bromley[5], B. Brown[5], D. Brunetti[8,5], R. Buckingham[5], M. Buckley[5], R. Budny, J. Buermans[4], H. Bufferand[10], P. Buratti[21], A. Burgess[5], A. Buscarino[31], A. Busse[5], D. Butcher[5], E. de la Cal[17], G. Calabrò[54], L. Calacci[55], R. Calado[11], Y. Camenen[111], G. Canal[56], B. Cannas[29], M. Cappelli[21], S. Carcangiu[29], P. Card[5], A. Cardinali[21], P. Carman[5], D. Carnevale[55], M. Carr[5], D. Carralero[17], L. Carraro[27], I.S. Carvalho[11], P. Carvalho[11], I. Casiraghi[7], F.J. Casson[5], C. Castaldo[21], J.P. Catalan[5], N. Catarino[11], F. Causa[8], M. Cavedon[6], M. Cecconello[22], C.D. Challis[5], B. Chamberlain[5], C.S. Chang[46], A. Chankin[6], B. Chapman[5,57], M. Chernyshova[58], A. Chiariello[16], P. Chmielewski[58], A. Chomiczewska[58], L. Chone[59], G. Ciraolo[10], D. Ciric[5], J. Citrin[60], Ł. Ciupinski[61], M. Clark[5], R. Clarkson[5], C. Clements[5], M. Cleverly[5], J.P. Coad[5], P. Coates[5], A. Cobalt[5], V. Coccorese[16], R. Coelho[11], J.W. Coenen[47], I.H. Coffey[62], A. Colangeli[21], L. Colas[10], C. Collins[32], J. Collins[5], S. Collins[5], D. Conka[28], S. Conroy[28], B. Conway[5], N.J. Conway[5], D. Coombs[5], P. Cooper[5], S. Cooper[5], C. Corradino[31], G. Corrigan[5], D. Coster[6], P. Cox[5], T. Craciunescu[63], S. Cramp[5], C. Crapper[5], D. Craven[5], R. Craven[5], M. Crialesi Esposito[51], G. Croci[7], D. Croft[5], A. Croitoru[63], K. Crombé[4,64], T. Cronin[5], N. Cruz[11], C. Crystal[35], G. Cseh[26], A. Cufar[9], A. Cullen[5], M. Curuia[65], T. Czarski[58], H. Dabirikhah[5], A. Dal Molin[7], E. Dale[5], P. Dalgliesh[5], S. Dalley[5], J. Dankowski[41], P. David[6], A. Davies[5], S. Davies[5], G. Davis[5], K. Dawson[5], S. Dawson[5], I.E. Day[5], M. De Bock[19], G. De Temmerman[19], G. De Tommasi[16], K. Deakin[5], J. Deane[5], R. Dejarnac[42], D. Del Sarto[38], E. Delabie[32], D. Del-Castillo-Negrete[32], A. Dempsey[66], R.O. Dendy[5,57], P. Devynck[10], A. Di Siena[6], C. Di Troia[21], T. Dickson[5], P. Dinca[63], T. Dittmar[47], J. Dobrashian[5], R.P. Doerner[67], A.J.H. Donné[68], S. Dorling[5], S. Dormido-Canto[69], D. Douai[10], S. Dowson[5], R. Doyle[66], M. Dreval[15,116], P. Drewelow[39], P. Drews[47], G. Drummond[5], Ph. Duckworth[19], H. Dudding[5,70], R. Dumont[10], P. Dumortier[4], D. Dunai[26], T. Dunatov[49], M. Dunne[6], I. Duran[42], F. Durodié[4], R. Dux[6], A. Dvornova[10], R. Eastham[5], J. Edwards[5], Th. Eich[6], A. Eichorn[5], N. Eidietis[35], A. Eksaeva[47], H. El Haroun[5], G. Ellwood[19], C. Elsmore[5], O. Embreus[71], S. Emery[5], G. Ericsson[22], B. Eriksson[22], F. Eriksson[72], J. Eriksson[22], L.G. Eriksson[73], S. Ertmer[47], S. Esquembri[25], A.L. Esquisabel[74], T. Estrada[17], G. Evans[5], S. Evans[5], E. Fable[6], D. Fagan[5], M. Faitsch[6], M. Falessi[21], A. Fanni[29], A. Farahani[5], I. Farquhar[5], A. Fasoli[43], B. Faugeras[48], S. Fazinić[49], F. Felici[43], R. Felton[5], A. Fernandes[11], H. Fernandes[11], J. Ferrand[5], D.R. Ferreira[11], J. Ferreira[11], G. Ferrò[55], J. Fessey[5], O. Ficker[42], A.R. Field[5], A. Figueiredo[11,33], J. Figueiredo[11,33], A. Fil[5], N. Fil[5,24], P. Finburg[5], D. Fiorucci[27], U. Fischer[34], G. Fishpool[5], L. Fittill[5], M. Fitzgerald[5], D. Flammini[21], J. Flanagan[5], K. Flinders[5], S. Foley[5], N. Fonnesu[21], M. Fontana[21], J.M. Fontdecaba[17], S. Forbes[5], A. Formisano[16], T. Fornal[58], L. Fortuna[31], E. Fortuna-Zalesna[61], M. Fortune[5], C. Fowler[5], E. Fransson[72], L. Frassinetti[37], M. Freisinger[47], R. Fresa[16], R. Fridström[37], D. Frigione[55], T. Fülöp[71], M. Furseman[5], V. Fusco[21], S. Futatani[75], D. Gadariya[17], K. Gál[68], D. Galassi[43], K. Gałązka[58], S. Galeani[55], D. Gallart[76], R. Galvão[27], Y. Gao[47], J. Garcia[10], M. García-Muñoz[77], M. Gardener[5], L. Garzotti[5], J. Gaspar[78], R. Gatto[79], P. Gaudio[55], D. Gear[5], T. Gebhart[32], S. Gee[5], M. Gelfusa[55], R. George[5], S.N. Gerasimov[5], G. Gervasini[20], M. Gethins[5], Z. Ghani[5], M. Gherendi[63], F. Ghezzi[8], J.C. Giacalone[10], L. Giacomelli[8], G. Giacometti[111], C. Gibson[5], K.J. Gibson[70], L. Gil[11], A. Gillgren[72], E. Giovannozzi[21], C. Giroud[5], R. Glen[5], S. Glöggler[6], J. Goff[5], P. Gohil[35], V. Goloborodko[80], R. Gomes[11], B. Gonçalves[11], M. Goniche[10], A. Goodyear[5], S. Gore[5], G. Gorini[7], T. Görler[6], N. Gotts[5], R. Goulding[46], E. Gow[5], B. Graham[5], J.P. Graves[43], H. Greuner[6], B. Grierson[46], J. Griffiths[5], S. Griph[5], D. Grist[5], W. Gromelski[58], M. Groth[59], R. Grove[32], M. Gruca[58], D. Guard[5], N. Gupta[5], C. Gurl[5], A. Gusarov[81], L. Hackett[5], S. Hacquin[10,33], R. Hager[46], L. Hägg[22], A. Hakola[14], M. Halitovs[28], S. Hall[5], S.A. Hall[5], S. Hallworth-Cook[5], C.J. Ham[5], D. Hamaguchi[2], M. Hamed[10], C. Hamlyn-Harris[5], K. Hammond[5], E. Harford[5], J.R. Harrison[5], D. Harting[5], Y. Hatano[82], D.R. Hatch[53], T. Haupt[5], J. Hawes[5], N.C. Hawkes[5], J. Hawkins[5], T. Hayashi[2], S. Hazael[5], S. Hazel[5],

P. Heesterman[5], W.W. Heidbrink[83], W. Helou[19], O. Hemming[5], S.S. Henderson[5], R.B. Henriques[11], D. Hepple[5], J. Herfindal[32], G. Hermon[5], J. Hill[5], J.C. Hillesheim[5], K. Hizanidis[30], A. Hjalmarsson[22], A. Ho[60], J. Hobirk[6], O. Hoenen[19], C. Hogben[5], A. Hollingsworth[5], S. Hollis[5], E. Hollmann[67], M. Hölzl[6], B. Homan[48], M. Hook[5], D. Hopley[5], J. Horáček[42], D. Horsley[5], N. Horsten[59], A. Horton[5], L.D. Horton[33,43], L. Horvath[5,70], S. Hotchin[5], R. Howell[5], Z. Hu[7], A. Huber[47], V. Huber[47], T. Huddleston[5], G.T.A. Huijsmans[19], P. Huynh[2], A. Hynes[5], D. Imrie[5], M. Imríšek[42], J. Ingleby[5], P. Innocente[27], K. Insulander Björk[71], N. Isernia[16], I. Ivanova-Stanik[58], E. Ivings[5], S. Jablonski[58], S. Jachmich[4,19,33], T. Jackson[5], P. Jacquet[5], H. Järleblad[84], F. Jaulmes[42], J. Jenaro Rodriguez[5], I. Jepu[63], E. Joffrin[10], R. Johnson[5], T. Johnson[37], J. Johnston[5], C. Jones[5], G. Jones[5], L. Jones[5], N. Jones[5], T. Jones[5], A. Joyce[5], R. Juarez[20], M. Juvonen[5], P. Kalnina[28], T. Kaltiaisenaho[14], J. Kaniewski[5], A. Kantor[5], A. Kappatou[6], J. Karhunen[13], D. Karkinsky[5], M. Kaufman[32], G. Kaveney[5], Ye.O. Kazakov[4], V.Kazantzidis[30], D.L. Keeling[5], R. Kelly[5], M. Kempenaars[19], C. Kennedy[5], D. Kennedy[5], J. Kent[5], K. Khan[5], C. Kiefer[6], J. Kilpeläinen[59], C. Kim[35], Hyun-Tae Kim[5,33], S.H. Kim[19], D.B. King[5], R. King[5], D. Kinna[5], V.G. Kiptily[5], A. Kirjasuo[14], K.K. Kirov[5], A. Kirschner[47], T. Kiviniemi[59], G. Kizane[28], M. Klas[85], C. Klepper[32], A. Klix[34], G. Kneale[5], M. Knight[5], P. Knight[5], R. Knights[5], S. Knipe[5], M. Knolker[35], S. Knott[86], M. Kocan[19], F. Köch[l9], I. Kodeli[9], Y. Kolesnichenko[80], Y. Kominis[30], M. Kong[5], V. Korovin[15], B. Kos[9], D. Kos[5], H.R. Koslowski[47], M. Kotschenreuther[53], M. Koubiti[111], E. Kowalska-Strzęciwilk[58], K. Koziol[12], V. Krasilnikov[19], M. Kresina[10,5], K. Krieger[6], N. Krishnan[5], A. Krivska[4], U. Kruezi[19], I. Książek[87], H. Kumpulainen[59], T. Kurki-Suonio[59], H. Kurotaki[2], S. Kwak[39], O.J. Kwon[88], L. Laguardia[8], E. Lagzdina[28], A. Lahtinen[13], A. Laing[5], N. Lam[5], H.T. Lambertz[47], B. Lane[5], C. Lane[5], E. Lascas Neto[43], E. Łaszyńska[58], K.D. Lawson[5], A. Lazaros[30], E. Lazzaro[8], G. Learoyd[5], Chanyoung Lee[89], S.E. Lee[82], S. Leerink[59], T. Leeson[5], X. Lefebvre[5], H.J. Leggate[66], J. Lehmann[5], M. Lehnen[19], D. Leichtle[34,90], F. Leipold[19], I. Lengar[9], M. Lennholm[5,73], E. Leon Gutierrez[17], B. Lepiavko[80], J. Leppänen[14], E. Lerche[4], A. Lescinskis[28], J. Lewis[5], W. Leysen[81], L. Li[47], Y. Li[47], J. Likonen[14], Ch. Linsmeier[47], B. Lipschultz[70], X. Litaudon[10,33], E. Litherland-Smith[5], F. Liu[10,33], T. Loarer[10], A. Loarte[19], R. Lobel[5], B. Lomanowski[32], P.J. Lomas[5], J.M. López[25], R. Lorenzini[27], S. Loreti[21], U. Losada[17], V.P. Loschiavo[16], M. Loughlin[19], Z. Louka[5], J. Lovell[32], T. Lowe[5], C. Lowry[5,73], S. Lubbad[5], T. Luce[19], R. Lucock[5], C. Luna[91], E. de la Luna[17], M. Lungaroni[55], C.P. Lungu[63], T. Lunt[6], V. Lutsenko[80], B. Lyons[35], A. Lyssoivan[4], M. Machielsen[43], E. Macusova[42], R. Mäenpää[59], C.F. Maggi[5], R. Maggiora[92], M. Magness[5], S. Mahesan[5], H. Maier[4], J. Mailloux[5], R. Maingi[46], K. Malinowski[58], P. Manas[111,22], P. Mantica[8], M.J. Mantsinen[93], J. Manyer[76], A. Manzanares[94], Ph. Maquet[19], G. Marceca[43], C. Marchetto[95], O. Marchuk[47], A. Mariani[8], G. Mariano[21], M. Marin[60], M. Marinelli[55], T. Markovič[42], D. Marocco[21], L. Marot[36], S. Marsden[5], J. Marsh[5], R. Marshall[5], L. Martellucci[55], A. Martin[5], A.J. Martin[5], R. Martone[16], S. Maruyama[19], M. Maslov[5], S. Masuzaki[23], S. Matejcik[34], M. Mattei[16], G.F. Matthews[5], D. Matveev[47], E. Matveeva[42], A. Mauriya[11], F. Maviglia[16], M. Mayer[4], M.-L. Mayoral[5,68], S. Mazzi[111,10], C. Mazzotta[21], R. McAdams[5], P.J. McCarthy[86], K.G. McClements[5], J. McClenaghan[35], P. McCullen[5], D.C. McDonald[5], D. McGuckin[5], D. McHugh[5], G. McIntyre[5], R. McKean[5], J. McKehon[5], B. McMillan[57], L. McNamee[5], A. McShee[5], A. Meakins[5], S. Medley[5], C.J. Meekes[60,96], K. Meghani[5], A.G. Meigs[5], G. Meisl[6], S. Meitner[32], S. Menmuir[5], K. Mergia[18], S. Merriman[5], Ph. Mertens[47], A. Messiaen[4], R. Michling[19], P. Middleton[5], D. Middleton-Gear[5], J. Mietelski[41], D. Milanesio[92], E. Milani[55], F. Militello[5], A. Militello Asp[5], J. Milnes[5], A. Milocco[7], G. Miloshevsky[97], C. Minghao[5], S. Minucci[54], I. Miron[63], M. Miyamoto[98], J. Mlynář[42,99], V. Moiseenko[15], P. Monaghan[5], I. Monakhov[5], T. Moody[5], S. Moon[37], R. Mooney[5], S. Moradi[4], J. Morales[10], R.B. Morales[5], S. Mordijck[100], L. Moreira[5], L. Morgan[5], F. Moro[21], J. Morris[5], K.-M. Morrison[5], L. Moser[19,36], D. Moulton[5], T. Mrowetz[5], T. Mundy[5], M. Muraglia[111], A. Murari[27,33], A. Muraro[8], N. Muthusonai[5], B. N'Konga[48], Yong-Su Na[89], F. Nabais[11], M. Naden[5], J. Naish[5], R. Naish[5], F. Napoli[21], E. Nardon[10], V. Naulin[84], M.F.F. Nave[11], I. Nedzelskiy[11], I. Nestoras[5], R. Neu[6], S. Ng[5], M. Nicassio[5], A.H. Nielsen[84], D. Nina[11], D. Nishijima[101], C. Noble[5], C.R. Nobs[5], M. Nocente[7,8], D. Nodwell[5], K. Nordlund[13], H. Nordman[72], R. Normanton[5], J.-M. Noterdaeme[6], S. Nowak[8], E. Nunn[5], H. Nyström[37], M. Oberparleiter[72], B. Obryk[41], J. O'Callaghan[5], T. Odupitan[5], H.J.C. Oliver[5,53], R. Olney[5], M. O'Mullane[102], J. Ongena[4], E. Organ[5], F. Orsitto[16], J. Orszagh[85], T. Osborne[35], R. Otin[5], T. Otsuka[103], A. Owen[5], Y. Oya[104], M. Oyaizu[2], R. Paccagnella[27], N. Pace[5], L.W. Packer[5], S. Paige[5], E. Pajuste[28], D. Palade[63], S.J.P. Pamela[5], N. Panadero[17], E. Panontin[7], A. Papadopoulos[30], G. Papp[6], P. Papp[85], V.V. Parail[5], C. Pardanaud[111], J. Parisi[5,105], F. Parra Diaz[105], A. Parsloe[5], M. Parsons[32], N. Parsons[5], M. Passeri[55], A. Patel[5], A. Pau[43], G. Pautasso[6], R. Pavlichenko[4], A. Pavone[39], E. Pawelec[5], C. Paz Soldan[106], A. Peacock[5,73], M. Pearce[5], E. Peluso[55], C. Penot[19], K. Pepperell[5], R. Pereira[11], T. Pereira[11], E. Perelli Cippo[8], P. Pereslavtsev[34], C. Perez von Thun[58], V. Pericoli[58], D. Perry[5], M. Peterka[42], P. Petersson[37], G. Petravich[26], N. Petrella[5], M. Peyman[5], M. Pillon[21], S. Pinches[19], G. Pintsuk[8], W. Pires de Sá[56], A. Pires dos Reis[56], C. Piron[21], L. Piron[27,107], A. Pironti[16], R. Pitts[19], K.L. van de Plassche[60], N. Platt[5], V. Plyusnin[11], M. Podesta[46], G. Pokol[26], F.M. Poli[46], O.G. Pompilian[63], S. Popovichev[5], M. Poradziński[58], M.T. Porfiri[21], M. Porkolab[24], C. Porosnicu[63], M. Porton[5], G. Poulipoulis[108], I. Predebon[27], G. Prestopino[55], C. Price[5], D. Price[5], M. Price[5], D. Primetzhofer[22], P. Prior[5], G. Provatas[49], G. Pucella[21], P. Puglia[43], K. Purahoo[5], I. Pusztai[71], O. Putignano[5], T. Pütterich[6], A. Quercia[16], E. Rachlew[71], G. Radulescu[32], V. Radulovic[9], M. Rainford[5], P. Raj[34], G. Ralph[5], G. Ramogida[21], D. Rasmussen[32], J.J. Rasmussen[84], G. Rattá[17], S. Ratynskaia[109], M. Rebai[8],

D. Réfy[26], R. Reichle[19], M. Reinke[32], D. Reiser[47], C. Reux[10], S. Reynolds[5], M.L. Richiusa[5], S. Richyal[5], D. Rigamonti[8], F.G. Rimini[5], J. Risner[32], M. Riva[21], J. Rivero-Rodriguez[77], C.M. Roach[5], R. Robins[5], S. Robinson[5], D. Robson[5], P. Rodrigues[11], M. Rodriguez Ramos[49], P. Rodriguez-Fernandez[24], F. Romanelli[21], M. Romanelli[5], S. Romanelli[5], J. Romazanov[47], R. Rossi[55], S. Rowe[5], D. Rowlands[5,33], M. Rubel[37], G. Rubinacci[16], G. Rubino[54], L. Ruchko[56], M. Ruiz[25], J. Ruiz Ruiz[105], C. Ruset[63], J. Rzadkiewicz[12], S. Saarelma[5], E. Safi[13], A. Sahlberg[22], M. Salewski[84], A. Salmi[14], R. Salmon[5], F. Salzedas[11,110], I. Sanders[5], D. Sandiford[5], B. Santos[11], A. Santucci[21], K. Särkimäki[71], R. Sarwar[5], I. Sarychev[5], O. Sauter[43], P. Sauwan[20], N. Scapin[51], F. Schluck[47], K. Schmid[6], S. Schmuck[8], M. Schneider[19], P.A. Schneider[6], D. Schwörer[66], G. Scott[5], M. Scott[5], D. Scraggs[5], S. Scully[5], M. Segato[5], Jaemin Seo[89], G. Sergienko[47], M. Sertoli[5], S.E. Sharapov[5], A. Shaw[5], H. Sheikh[5], U. Sheikh[43], A. Shepherd[5], P. Shigin[19], K. Shinohara[3], S. Shiraiwa[46], D. Shiraki[32], M. Short[5], G. Sias[29], S.A. Silburn[5], A. Silva[11], C. Silva[11], J. Silva[5], D. Silvagni[6], D. Simfukwe[5], J. Simpson[5,59], D. Sinclair[5], S.K. Sipilä[59], A.C.C. Sips[73], P. Sirén[13], A. Sirinelli[19], H. Sjöstrand[22], N. Skinner[5], J. Slater[5], N. Smith[5], P. Smith[5], J. Snell[5], G. Snoep[60], L. Snoj[9], P. Snyder[35], S. Soare[65], E.R. Solano[17], V. Solokha[59], A. Somers[66], C. Sommariva[43], K. Soni[36], E. Sorokovoy[15], M. Sos[42], J. Sousa[11], C. Sozzi[8], S. Spagnolo[27], T. Spelzini[5], F. Spineanu[63], D. Spong[32], D. Sprada[5], S. Sridhar[10], C. Srinivasan[5], G. Stables[5], G. Staebler[35], I. Stamatelatos[18], Ž. Štancar[9,5], P. Staniec[5], G. Stankunas[112], M. Stead[5], E. Stefanikova[37], A. Stephen[5], J. Stephens[5], P. Stevenson[5], M. Stojanov[5], P. Strand[72], H.R. Strauss[113], S. Strikwerda[5], P. Ström[37], C.I. Stuart[5], W. Studholme[5], M. Subramani[5], E. Suchkov[85], S. Sumida[2], H.J. Sun[5], T.E. Susts[28], J. Svensson[39], J. Svoboda[42], R. Sweeney[24], D. Sytnykov[15], T. Szabolics[26], G. Szepesi[5], B. Tabia[5], T. Tadić[49], B. Tál[6], T. Tala[14], A. Tallargio[5], P. Tamain[10], H. Tan[5], K. Tanaka[23], W. Tang[46], M. Tardocchi[8], D. Taylor[5], A.S. Teimane[28], G. Telesca[58], A. Teplukhina[46], D. Terentyev[81], A. Terra[47], D. Terranova[27], N. Terranova[21], D. Testa[43], E. Tholerus[5,37], J. Thomas[5], E. Thoren[109], A. Thorman[5], W. Tierens[6], R.A. Tinguely[24], A. Tipton[5], H. Todd[5], M. Tokitani[23], P. Tolias[109], M. Tomeš[42], A. Tookey[5], Y. Torikai[114], U. von Toussaint[6], P. Tsavalas[18], D. Tskhakaya[42,115], I. Turner[5], M. Turner[5], M.M. Turner[66], M. Turnyanskiy[5,68], G. Tvalashvili[5], S. Tyrrell[5], M. Tyshchenko[80], A. Uccello[8], V. Udintsev[19], G. Urbanczyk[10], A. Vadgama[5], D. Valcarcel[5], M. Valisa[27], P. Vallejos Olivares[37], O. Vallhagen[71], M. Valovič[5], D. Van Eester[4], J. Varje[59], S. Vartanian[10], T. Vasilopoulou[18], G. Vayakis[19], M. Vecsei[26], J. Vega[17], S. Ventre[16], G. Verdoolaege[64], C. Verona[55], G. Verona Rinati[55], E. Veshchev[19], N. Vianello[27], E. Viezzer[77], L. Vignitchouk[109], R. Vila[17], R. Villari[21], F. Villone[16], P. Vincenzi[27], B. Viola[21], A.J. Virtanen[59], A. Vitins[28], Z. Vizvary[5], G. Vlad[21], M. Vlad[63], P. Vondráček[42], P. de Vries[19], B. Wakeling[5], N.R. Walkden[5], M. Walker[5], R. Walker[5], M. Walsh[19], E. Wang[47], N. Wang[5], S. Warder[5], R. Warren[5], J. Waterhouse[5], C. Watts[19], T. Wauters[4], A. Weckmann[37], H. Wedderburn Maxwell[5], M. Weiland[6], H. Weisen[43], M. Weiszflog[22], P. Welch[5], N. Wendler[58], A. West[5], M. Wheatley[5], S. Wheeler[5], A. Whitehead[5], D. Whittaker[5], A. Widdowson[5], S. Wiesen[47], J. Wilkinson[5], J.C. Williams[5], D. Willoughby[5], I. Wilson[5], J. Wilson[5], T. Wilson[5], M. Wischmeier[6], P. Wise[5], G. Withenshaw[5], A. Withycombe[5], D. Witts[5], A. Wojcik-Gargula[41], E. Wolfrum[6], R. Wood[5], C. Woodley[5], R. Woodley[5], B. Woods[5], J. Wright[5], J.C. Wright[24], T. Xu[5], D. Yadikin[72], M. Yajima[23], Y. Yakovenko[80], Y. Yang[19], W. Yanling[47], V. Yanovskiy[42], I. Young[5], R. Young[5], R.J. Zabolockis[28], J. Zacks[5], R. Zagorski[12], F.S. Zaitsev[85], L. Zakharov[13], A. Zarins[28], D. Zarzoso Fernandez[111], K.-D. Zastrow[5], Y. Zayachuk[5], M. Zerbini[21], W. Zhang[6], Y. Zhou[37], M. Zlobinski[47], A. Zocco[39], A. Zohar[9], V. Zoita[63], S. Zoletnik[26], V.K. Zotta[79], I. Zoulias[5], W. Zwingmann[11] & I. Zychor[12]

[1] National Institutes for Quantum Science and Technology (QST), Rokkasho Fusion Institute, Rokkasho, Aomori 039-3212, Japan
[2] National Institutes for Quantum Science and Technology (QST), Naka Fusion Institute, Naka, Ibaraki 311-0193, Japan
[3] The University of Tokyo, Kashiwa, Chiba, 277-0882, Japan
[4] Laboratory for Plasma Physics, LPP-ERM/KMS, TEC Partner, BE-1000 Brussels, Belgium
[5] United Kingdom Atomic Energy Authority, Culham Science Centre, Abingdon, Oxon, OX14 3DB, United Kingdom
[6] Max-Planck-Institut für Plasmaphysik, D-85748 Garching, Germany
[7] University of Milano-Bicocca, Piazza della Scienza 3, 20126 Milano, Italy
[8] Institute for Plasma Science and Technology, CNR, via R. Cozzi 53, 20125 Milano, Italy
[9] Slovenian Fusion Association (SFA), Jožef Stefan Institute, Jamova cesta 39, SI-1000 Ljubljana, Slovenia
[10] CEA, IRFM, F-13108 Saint Paul Lez Durance, France
[11] Instituto de Plasmas e Fusão Nuclear, Instituto Superior Técnico, Universidade de Lisboa, 1049-001 Lisboa, Portugal
[12] National Centre for Nuclear Research (NCBJ), 05-400 Otwock-Świerk, Poland
[13] University of Helsinki, PO Box 43, FI-00014 University of Helsinki, Finland
[14] VTT Technical Research Centre of Finland, PO Box 1000, FIN-02044 VTT, Finland
[15] Institute of Plasma Physics, National Science Center, Kharkiv Institute of Physics and Technology, Akademichna 1, Kharkiv 61108, Ukraine
[16] Consorzio CREATE, Via Claudio 21, 80125 Napoli, Italy

[17] Laboratorio Nacional de Fusión, CIEMAT, Madrid, Spain
[18] NCSR 'Demokritos' 15310, Agia Paraskevi Attikis, Greece
[19] ITER Organization, Route de Vinon-sur-Verdon, CS 90 046, 13067 Saint Paul Lez Durance Cedex, France
[20] Universidad Nacional de Educacion a Distancia, Dept Ingn Energet, Calle Juan del Rosal 12, E-28040 Madrid, Spain
[21] Dipartimento Fusione e Tecnologie per la Sicurezza Nucleare, ENEA C. R. Frascati, via E. Fermi 45, 00044 Frascati (Roma), Italy
[22] Department of Physics and Astronomy, Uppsala University, SE-75120 Uppsala, Sweden
[23] National Institute for Fusion Science, Oroshi, Toki, Gifu 509-5292, Japan
[24] MIT Plasma Science and Fusion Center, Cambridge, MA 02139, United States of America
[25] Universidad Politécnica de Madrid, Grupo I2A2, Madrid, Spain
[26] Centre for Energy Research, POB 49, H-1525 Budapest, Hungary
[27] Consorzio RFX, Corso Stati Uniti 4, 35127 Padova, Italy
[28] University of Latvia, 19 Raina Blvd., Riga, LV 1586, Latvia
[29] Department of Electrical and Electronic Engineering, University of Cagliari, Piazza d'Armi, 09123 Cagliari, Italy
[30] National Technical University of Athens, Iroon Politechniou 9, 157 73 Zografou, Athens, Greece
[31] Dipartimento di Ingegneria Elettrica Elettronica e Informatica, Università degli Studi di Catania, 95125 Catania, Italy
[32] Oak Ridge National Laboratory, Oak Ridge, TN 37831, TN, United States of America
[33] EUROfusion Programme Management Unit, Culham Science Centre, Culham, OX14 3DB, United Kingdom
[34] Karlsruhe Institute of Technology, PO Box 3640, D-76021 Karlsruhe, Germany
[35] General Atomics, PO Box 85608, San Diego, CA 92186-5608, United States of America
[36] Department of Physics, University of Basel, Switzerland
[37] Fusion Plasma Physics, EECS, KTH Royal Institute of Technology, SE-10044 Stockholm, Sweden
[38] Institut Jean Lamour, UMR 7198, CNRS-Université de Lorraine, 54500 Vandoeuvre-lès-Nancy, France
[39] Max-Planck-Institut für Plasmaphysik, Teilinstitut Greifswald, D-17491 Greifswald, Germany
[40] Maritime University of Szczecin Faculty of Marine Engineering, Waly Chrobrego 1-2, 70-500 Szczecin, Poland
[41] Institute of Nuclear Physics, Radzikowskiego 152, 31-342 Kraków, Poland
[42] Institute of Plasma Physics of the CAS, Za Slovankou 1782/3, 182 00 Praha 8, Czech Republic
[43] Ecole Polytechnique Fédérale de Lausanne (EPFL), Swiss Plasma Center (SPC), CH-1015 Lausanne, Switzerland
[44] University of Wisconsin-Madison, Madison, WI 53706, United States of America
[45] Magnetic Sensor Laboratory, Lviv Polytechnic National University, Lviv, Ukraine
[46] Princeton Plasma Physics Laboratory, James Forrestal Campus, Princeton, NJ 08543, NJ, United States of America
[47] Forschungszentrum Jülich GmbH, Institut für Energie- und Klimaforschung, Plasmaphysik, 52425 Jülich, Germany
[48] Université Cote d'Azur, CNRS, Inria, LJAD, Parc Valrose, 06108 Nice Cedex 02, France
[49] Ruđer Bošković Institute, Bijenička 54, 10000 Zagreb, Croatia
[50] The National Institute for Optoelectronics, Magurele-Bucharest, Romania
[51] Mechanics, SCI, KTH SE-100 44 Stockholm, Sweden
[52] Fourth State Research, 503 Lockhart Dr, Austin, TX, United States of America
[53] University of Texas at Austin, Institute for Fusion Studies, Austin, TX 78712, United States of America
[54] University of Tuscia, DEIM, Via del Paradiso 47, 01100 Viterbo, Italy
[55] Università di Roma Tor Vergata, Via del Politecnico 1, Roma, Italy
[56] Instituto de Física, Universidade de São Paulo, Rua do Matão Travessa R Nr.187, CEP 05508-090 Cidade Universitária, São Paulo, Brasil
[57] Centre for Fusion, Space and Astrophysics, University of Warwick, Coventry, CV4 7AL, United Kingdom
[58] Institute of Plasma Physics and Laser Microfusion, Hery 23, 01-497 Warsaw, Poland
[59] Aalto University, PO Box 14100, FIN-00076 Aalto, Finland
[60] FOM Institute DIFFER, Eindhoven, Netherlands
[61] Warsaw University of Technology, 02-507 Warsaw, Poland
[62] Astrophysics Research Centre, School of Mathematics and Physics, Queen's University, Belfast, BT7 1NN, United Kingdom
[63] The National Institute for Laser, Plasma and Radiation Physics, Magurele-Bucharest, Romania
[64] Department of Applied Physics, Ghent University, 9000 Ghent, Belgium
[65] The National Institute for Cryogenics and Isotopic Technology, Ramnicu Valcea, Romania
[66] Dublin City University (DCU), Dublin, Ireland
[67] University of California at San Diego, La Jolla, CA 92093, United States of America
[68] EUROfusion Programme Management Unit, Boltzmannstr. 2, 85748 Garching, Germany
[69] UNED, Dpto. Informática y Automática, Madrid, Spain

[70] York Plasma Institute, Department of Physics, University of York, York, YO10 5DD, United Kingdom
[71] Department of Physics, Chalmers University of Technology, SE-41296 Gothenburg, Sweden
[72] Department of Space, Earth and Environment, Chalmers University of Technology, SE-41296 Gothenburg, Sweden
[73] European Commission, B-1049 Brussels, Belgium
[74] University of Tennessee, Knoxville, TN 37996, TN, United States of America
[75] Universitat Politècnica de Catalunya, Barcelona, Spain
[76] Barcelona Supercomputing Center, Barcelona, Spain
[77] Universidad de Sevilla, Sevilla, Spain
[78] Aix-Marseille University, CNRS, IUSTI, UMR 7343, 13013 Marseille, France
[79] Dipartimento di Ingegneria Astronautica, Elettrica ed Energetica, SAPIENZA Università di Roma, Via Eudossiana 18, 00184 Roma, Italy
[80] Institute for Nuclear Research, Prospekt Nauky 47, Kyiv 03680, Ukraine
[81] Studiecentrum voor Kernenergie - Centre d'Etude de l'Energie Nucléaire, Boeretang 200, 2400 Mol, Belgium
[82] University of Toyama, Toyama, 930-8555, Japan
[83] University of California, Irvine, Irvine, California 92697, United States of America
[84] Department of Physics, Technical University of Denmark, Bldg 309, DK-2800 Kgs Lyngby, Denmark
[85] Faculty of Mathematics, Department of Experimental Physics, Physics and Informatics Comenius University Mlynska dolina F2, 84248 Bratislava, Slovakia
[86] University College Cork (UCC), Cork, Ireland
[87] Institute of Physics, Opole University, Oleska 48, 45-052 Opole, Poland
[88] Daegu University, Jillyang, Gyeongsan, Gyeongbuk 712-174, Republic of Korea
[89] Department of Nuclear Engineering, Seoul National University, Seoul, South Korea
[90] Fusion for Energy Joint Undertaking, Josep Pl. 2, Torres Diagonal Litoral B3, 08019, Barcelona, Spain
[91] Arizona State University, Tempe, AZ, United States of America
[92] Politecnico di Torino, Corso Duca degli Abruzzi 24, I-10129 Torino, Italy
[93] ICREA and Barcelona Supercomputing Center, Barcelona, Spain
[94] Universidad Complutense de Madrid, Madrid, Spain
[95] Istituto dei Sistemi Complessi - CNR and Dipartimento di Energia - Politecnico di Torino, Corso Duca degli Abruzzi 24, 10129 Torino, Italy
[96] Eindhoven University of Technology, Netherlands
[97] Purdue University, 610 Purdue Mall, West Lafayette, IN 47907, United States of America
[98] Department of Material Science, Shimane University, 1060 Nishikawatsu, Matsue, 690-8504, Japan
[99] Faculty of Nuclear Sciences and Physical Engineering, Czech Technical University in Prague, Břehová 78/7, 115 19 Praha 1, Czech Republic
[100] College of William and Mary, Williamsburg, VA 23185, United States of America
[101] University of California, 1111 Franklin St., Oakland, CA 94607, United States of America
[102] University of Strathclyde, Glasgow, G4 0NG, United Kingdom
[103] Kindai University, Higashi-Osaka, 577-8502, Japan
[104] Shizuoka University, Shizuoka, 422-8529, Japan
[105] Rudolf Peierls Centre for Theoretical Physics, University of Oxford, Oxford OX1 3PU, United Kingdom
[106] Columbia University, New York, NY 10027, United States of America
[107] Dipartimento di Fisica "G. Galilei", Università degli Studi di Padova, Padova, Italy
[108] University of Ioannina, Panepistimioupoli Ioanninon, PO Box 1186, 45110 Ioannina, Greece
[109] Space and Plasma Physics, EECS, KTH SE-100 44 Stockholm, Sweden
[110] Universidade do Porto, Faculdade de Engenharia, 4200-465 Porto, Portugal
[111] Aix-Marseille University, CNRS, PIIM, UMR 7345, 13013 Marseille, France
[112] Lithuanian Energy Institute, Breslaujos g. 3, LT-44403, Kaunas, Lithuania
[113] HRS Fusion, West Orange, NJ, United States of America
[114] Ibaraki University Graduate School of Science and Engineering, Mito, Ibaraki 310-8512, Japan
[115] Technische Universität Wien, Fusion@ÖAW Österreichische Akademie der Wissenschaften (ÖAW), Austria
[116] V.N. Karazin Kharkiv National University, Kharkiv, Ukraine
